# Synthesis, Anti-Tumour, and Antibacterial Activities of Monocarbonyl Curcumin Analogues of Piperidones

**DOI:** 10.3390/ijms262412179

**Published:** 2025-12-18

**Authors:** Renhua Dong, Ruirui Xu, Xiuli Wang

**Affiliations:** School of Chinese Materia Medica, Beijing University of Chinese Medicine, Beijing 102488, China; o1359795489@163.com (R.D.); 18801096361@163.com (R.X.)

**Keywords:** curcumin, analogues, anticancer, antitumor, methicillin-resistant *Staphylococcus aureus* (MRSA)

## Abstract

Curcumin has anti-tumour and antibacterial effects. In this research, fourteen kinds of piperidone monocarbonyl curcumin analogues with 3,5-dimethylene-4-piperidone as the parent scaffold and halogen substitution on both sides of the benzene ring were synthesized by Claisen–Schmidt reaction, and their anti-tumour effect, mechanism, and antibacterial activity were investigated. It was found that a series of curcumin analogues has different degrees of anti-tumour and antibacterial dual activity. Among them, 2,5-2Cl, 2Br-5Cl, 2-Cl, 2-F, and benzaldehyde have strong broad-spectrum anti-tumour effects and have obvious selective inhibitory effects on A549 cells. The IC_50_ value is less than 5 μmol/L. The five promising compounds, respectively, inhibited the expression of AKT and ERK to induce apoptosis of A549 cells to varying degrees. The newly synthesized analogues 2,5-2Cl and 2Br-5Cl had stronger inhibitory effects on the growth of A549 cells than other analogues, and they tended to mainly inhibit the expression of AKT and ERK, respectively. However, 2-Cl and 2-F have significantly better inhibitory effects on methicillin-resistant *Staphylococcus aureus* (MRSA) than antibiotics. Taken together, piperidone monocarbonyl curcumin analogues may be developed as good candidates for potential prevention and treatment of cancer and bacterial infection complications.

## 1. Introduction

With the rapid development of medical options, the survival rate of cancer has been significantly improved. However, cancer remains a major global public health problem and the second leading cause of death in the United States. In 2023, the United States alone is reported to have had 1,958,310 new cancer cases, of which there were 609,820 deaths. Among them, lung cancer is the foremost fatal cancer and the leading cause of cancer death in men and women aged 50 and over. About 350 people die of lung cancer every day. Breast cancer is the leading cause of death in women aged 20–79 years, and cervical cancer is the second leading cause of cancer death in women aged 20–39 years. Hepatocellular carcinoma (HCC) is one of the most common malignant tumours in the world, with the second-lowest 5-year survival rate (21%) [1]. Chemotherapy is still considered to be the most effective method for the treatment of cancer, but current chemotherapy drugs usually act on a single target, and the treatment effect is not good. Among them, curcumin is one of the most studied multi-target drugs. It has preventive and therapeutic value for a variety of cancers, including prostate cancer, breast cancer, cervical cancer, liver cancer, colon cancer, and pancreatic cancer [2,3]. It is listed as the third-generation cancer chemopreventive drug by the National Cancer Institute [4]. In addition, infection is a global medical challenge. Methicillin-resistant *Staphylococcus aureus* (MRSA) is one of the clinical ‘superbugs’. It has multiple drug resistances to traditional antibiotics such as penicillin and its derivatives, which can lead to life-threatening infections, such as skin and soft tissue infections, blood infections, and systemic organ infections, causing sepsis, infective endocarditis, osteomyelitis, pneumonia, lung abscess, meningitis, and other serious diseases. It is an important pathogen causing death in hospitals and community infections. Therefore, it is necessary to develop more effective and safe treatments to prevent infection, and curcumin also has the potential to serve as a basis for developing powerful antibiotics to treat MRSA [5,6].

**Figure 1:**
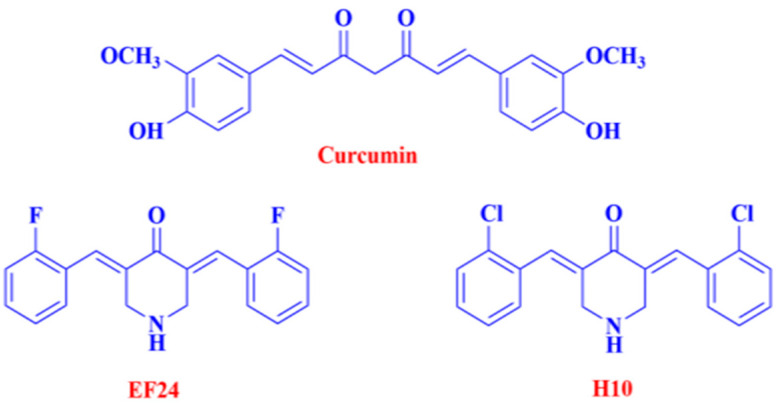
Chemical structures of curcumin, EF24, and H10.

Curcumin is a natural phenolic compound extracted and isolated from the rhizome of turmeric plants [7,8]. Current studies have found that curcumin has anti-tumour, antibacterial, anti-inflammatory, anti-oxidation, cardiovascular protection, and other pharmacological effects [9,10,11]. However, in practical applications, curcumin’s low solubility in vivo, rapid metabolism, short half-life, low bioavailability, and the requirement for multiple long-term oral administrations significantly limit its clinical application [12,13,14,15]. One of the main reasons for the high instability, rapid degradation, and low bioavailability of curcumin is the presence of β-diketone in the structure. The β-diketone part of curcumin is a specific substrate of hepatic aldosterone reductase, which can lead to the rapid metabolism of curcumin in vivo [16,17,18]. Therefore, many researchers have carried out a series of modifications, including modifications on the β-diketone structure of curcumin. It has been found that when the β-diketone structure is replaced by a monocarbonyl group, in addition to enhancing the stability significantly, it can also improve its pharmacological activity. The biological activity of monocarbonyl curcumin derivatives mainly focuses on anti-tumour, anti-inflammatory, and antibacterial aspects [19,20,21]. Among them, piperidone monocarbonyl curcumin analogues have been studied more [22]. Studies have shown that the introduction of piperidone can inhibit the growth of colon adenocarcinoma cell Caco-2 and human neuroblastoma cell SH-SY5Y after replacing the β-diketone structure of curcumin with 4-piperidone [23]. The β-diketone structure of curcumin was modified into a five-membered nitrogen-containing heterocyclic ring, resulting in piperidone monocarbonyl curcumin analogues. Its representative compound 3,5-bis(2-fluorobenzylidene)-4-piperidone (EF24) can not only effectively inhibit a variety of tumour cells but also inhibit the growth of Gram-negative bacteria such as *Escherichia coli* and Gram-positive bacteria such as *Mycobacterium tuberculosis* and *Staphylococcus aureus* [24]. Compound 3,5-bis(2-chlorobenzylidene)-4-piperidone (H10) inhibits prostate cancer by inhibiting 17β-hydroxysteroid dehydrogenase type 3 (17β-HSD3) [25,26]. Based on the above research results, we found that the activity of the modified curcumin analogues was greatly increased. By modifying compound EF24, its analogue 3,5-bis(2-bromobenzylidene)piperidine-4-one (B6) was synthesized. It was found that B6 had a higher inhibitory effect on tumour cells than compound EF24. Based on the above research results, we found that the activity of the modified curcumin analogues was greatly increased [25]. Compounds EF24 and H10 (Figure 1) have the characteristics of 3,5-dimethylene-4-piperidone as the parent nucleus and halogen substitution on both sides of the benzene ring, which stimulates our interest in synthesizing such derivatives and finding compounds with anti-tumour or antibacterial pharmacological activity.

In this study, a series of halogen-substituted piperidone monocarbonyl curcumin analogues were synthesized by chemical reaction, and their anti-tumour activity and structure–activity relationship were studied. In addition, the antibacterial activity against MRSA was also evaluated in order to explore curcumin derivatives with dual biological anti-tumourtumor and antibacterial activitiestumour, and to provide some basic guidance for further chemical research or pharmaceutical development.

## 2. Results and Discussion

### 2.1. Chemistry

**Scheme 1:**
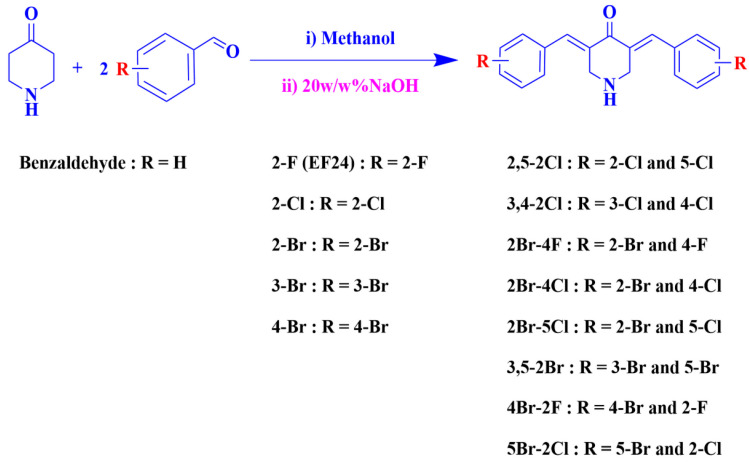
Synthesis of piperidone monocarbonyl curcumin analogues.

Through the Claisen–Schmidt reaction, 14 compounds were synthesized, similar to EF24. The structure was confirmed by ^1^H-NMR, ^13^C-NMR, and EI-MS. The synthetic route and structure of the target compound are shown in Scheme 1. The specific reaction mechanism is as follows: The condensation reaction of aldehydes without α-H with aliphatic aldehydes or ketones containing α-H occurs in the alcohol solution of dilute sodium hydroxide, and the product is dehydrated to obtain α, β-unsaturated aldehydes or ketones with high yield. The peak of heavy water was found in the nuclear magnetic spectrum characterized by compounds, and the chemical shift of hydrogen on the secondary amine was in the 2.0 space. After the protonation of heavy water, the shift changed, so there was no peak at δ (ppm) 2.0. Otherwise, ^1^H-NMR, ^13^C-NMR, and HR-MS diagrams of 14 compounds are shown in the Supplementary Materials.

The Claisen–Schmidt rearrangement reaction can react under both acidic and alkaline conditions. Combined with literature reports and actual test results [27,28,29], this study chose to take place with the synthesis reaction under alkaline conditions and neutralize it with acid until the system became neutral. After multiple experiments, it was found that the temperature and the pH of the reaction fluid would affect the yield of the synthesis. The synthesis yield was higher at room temperature, 18–20 °C, and lower at room temperatures above 25 °C. When the reaction system was too alkaline, it was easy to form large cohesive caking and more by-products with lower synthesis efficiency. The synthesis rate was also greatly reduced when too much acid was added. At times 2 h and 2.5 h of the reaction process, a small amount of reaction fluid was extracted, and there was no difference in thin-layer detection, indicating that the reaction time was 2 h.

**Figure 2:**
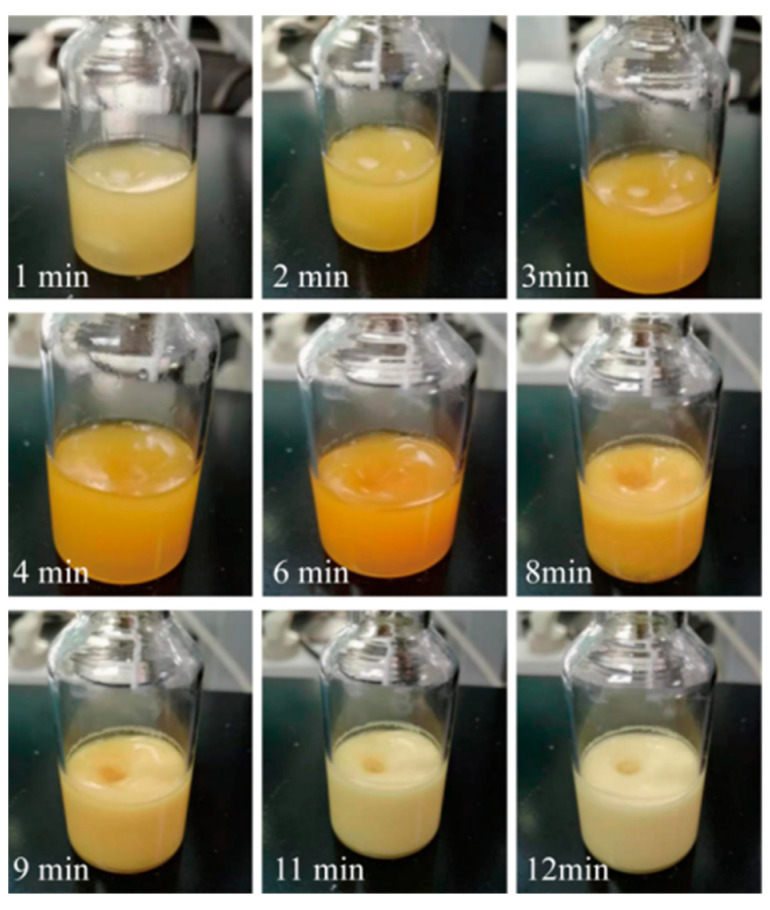
The colour change process of the synthesis reaction of piperidone monocarbonyl curcumin analogues.

Due to the colour of the system changing during the reaction, the process could be determined not only by TLC detection, but also by the colour of the reaction liquid. After alkali was added to the reaction fluid, the colour change over time is shown in Figure 2, successively 1 min, 2 min, 3 min, 4 min, 6 min, 8 min, 9 min, 11 min, and 12 min. The solution colour was speculated to be related to the amount of alkali added, as the more alkali added, the darker the colour of the reaction solution. As the reaction went on, the enol structure was dehydrated to form β-unsaturated ketones, and the colour of the reaction solution became lighter.

### 2.2. Biological Evaluation

#### 2.2.1. Anti-Tumour Activity

##### Piperidone Monocarbonyl Curcumin Analogues Inhibited the Proliferation of Tumour Cell Lines In Vitro

MTT assay was used to preliminarily evaluate the inhibitory effects of curcumin and fourteen piperidone monocarbonyl curcumin analogues, as well as cisplatin solution as a positive control, on the growth of HepG2 cells, A549 cells, Hela cells, and MCF-7 cells, and IC_50_ was calculated. The test results were shown in Table 1. The IC_50_ of fourteen piperidone monocarbonyl curcumin analogues to one or more of the four tumour cell lines was lower than that of curcumin and cisplatin solution, with a stronger inhibitory effect on tumour cell growth. Among them, benzaldehyde, 2,5-2Cl, 2-F (EF24), 2-Cl, and 2Br-5Cl, five piperidone monocarbonyl curcumin analogues, had stronger inhibitory effects on the growth of four tumour cell lines, with IC_50_ values below 15 μmol/L, most of which were below 10 μmol/L.

Compared with HepG2 cells, Hela cells, and McF-7 cells, the IC_50_ of the five promising compounds on A549 cells was smaller. Therefore, the five piperidone monocarbonyl curcumin analogues were selected for subsequent biological activity studies on the A549 cell line.

##### Five Promising Compounds Inhibited the Growth of A549 Cells

**Figure 3:**
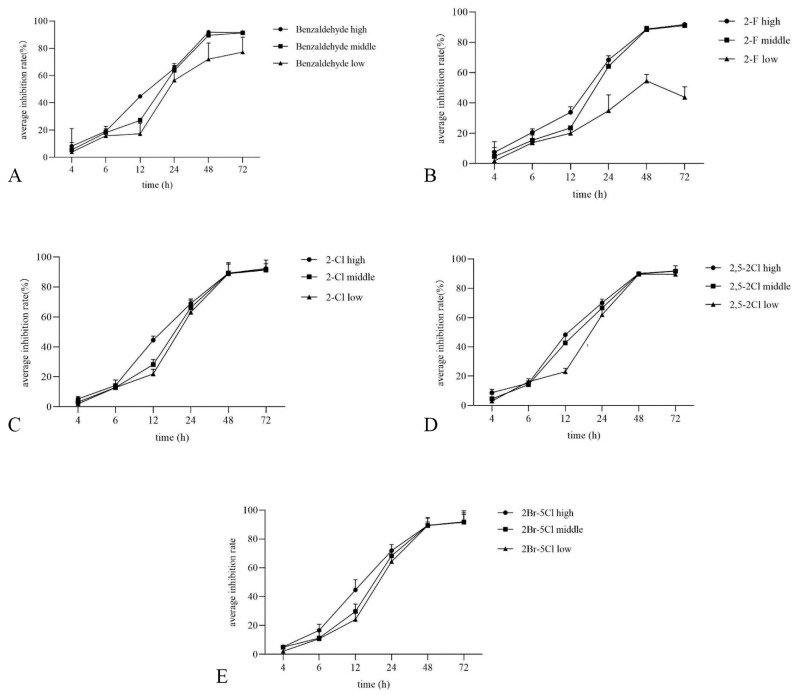
The growth inhibition curve of five promising monocarbonyl curcumin analogues on A549 cells. (**A**) Benzaldehyde group; (**B**) 2-F group; (**C**) 2-Cl group; (**D**) 2,5-2Cl group; and (**E**) 2Br-5Cl group. ***Note:*** *Standard deviation of some groups is less than 1, so it cannot be reflected in the line chart*.

In this study, the CCK-8 method was used to measure the pharmacodynamics and time action curves of five promising compounds at different concentrations on A549 cells, as shown in Figure 3.

The inhibitory effects of the five promising compounds on the growth of A549 cells were dose-dependent and time-dependent. The inhibition rate of the five promising compounds on A549 cells increased significantly with the prolongation of the drug action time. The inhibition rate of the five promising piperidone monocarbonyl curcumin analogues on A549 cells either increased slowly, remained unchanged, or even decreased after 48 h of drug treatment. In the early stage of this experiment, the inhibitory effects of fourteen curcumin analogues on four types of tumour cells were determined for 24 h. Therefore, in the subsequent experimental exploration, in order to ensure the comparability of experimental results and the uniformity of the experiment, the drug action time was set at 24 h.

##### Toxicity of Five Promising Compounds to Human Normal Liver Cells (L02)

The MTT method was used to detect the toxicity of five promising compounds to L02 cells, and the results are shown in Table 2. Compared with Table 1, IC_50_ values of the five promising compounds on L02 cells were much higher than their toxicity on tumour cells.

The IC_50_ of 2,5-2Cl, 2-F, 2-Cl, 2Br-5Cl, and benzaldehyde on L02 cells were 48.59 μmol/L, 62.03 μmol/L, 70.48 μmol/L, 58.25 μmol/L, and 52.36 μmol/L, respectively, which were about 16.9 times, 15.5 times, 14.7 times, 12.3 times, and 11.2 times of A549 cells. The IC_50_ of curcumin and cisplatin solution on L02 cells was 30.40 μmol/L and 48.36 μmol/L, which were about 1.2 times and 0.9 times that of A549 cells.

The results showed that the five promising compounds had obvious selectivity to A549 cells between L02 cells and A549 cells, and the selectivity of the five promising compounds to A549 cells was significantly better than the positive drug cisplatin and curcumin.

##### Effects of Five Promising Compounds on A549 Cell Migration

**Figure 4:**
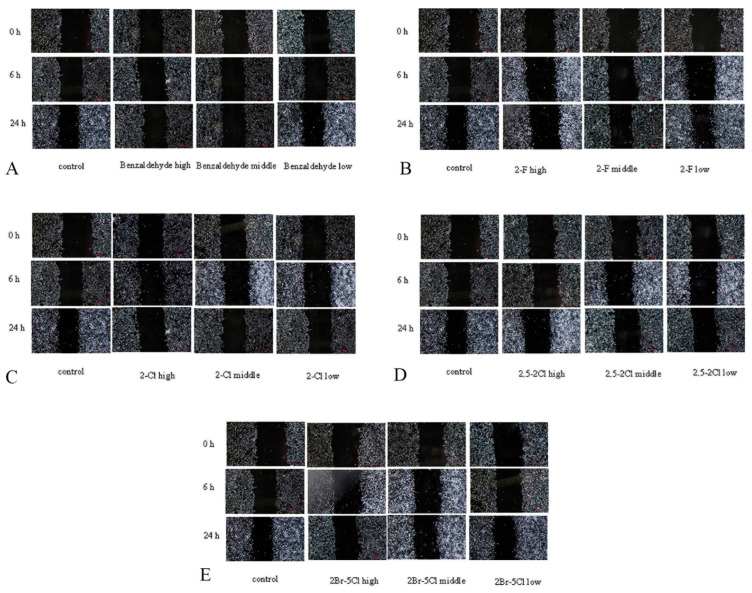
The effect of five monocarbonyl curcumin analogues on the migration of A549 cells (80×). (**A**) Benzaldehyde group; (**B**) 2-F group; (**C**) 2-Cl group; (**D**) 2,5-2Cl group; and (**E**) 2Br-5Cl group. ***Note:*** 2,5-2Cl: High, medium, and low drug concentrations are 2.5 μmol/L, 1.25 μmol/L, and 0.625 μmol/L, respectively. 2Br-5Cl, 2-Cl, 2-F, benzaldehyde: High, medium, and low drug concentrations are 5 μmol/L, 2.5 μmol/L, and 1.25 μmol/L, respectively.

**Figure 5:**
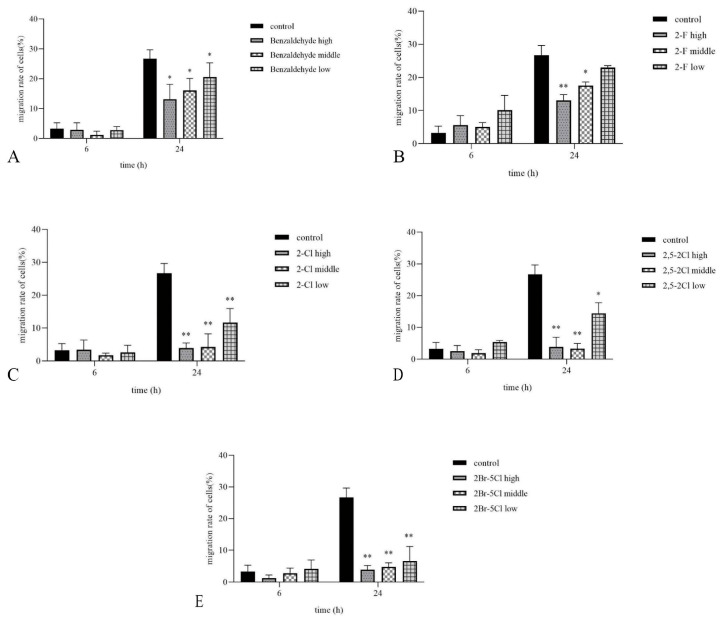
The effect of five monocarbonyl curcumin analogues on the migration distance of A549 cells. (**A**) Benzaldehyde group; (**B**) 2-F group; (**C**) 2-Cl group; (**D**) 2,5-2Cl group; and (**E**) 2Br-5Cl group. **Note:** Compared with blank control group (0.9% DMSO), * p < 0.05; ** p < 0.01.

In this study, the inhibition effects of five promising compounds on A549 cell migration were qualitatively evaluated by cell wound scratch assay. Figure 4 shows the comparison of scratch distance of A549 cells after 0 h, 6 h, and 24 h intervention with different compounds, and Figure 5 shows the histogram of the ratio of scratch area of A549 cells at 6 h and 24 h after treatment with five different promising compounds at different concentrations.

Compared with 0 h, the cells in the blank control group showed obvious migration at 24 h. Scratches showed growth and healing, with sufficient migration area and good healing, indicating that A549 cells had migration characteristics without adding drugs. With the increase in drug concentration in the drug group, the scratch healing of A549 cells became worse and the migration area became smaller, indicating that the five promising compounds could inhibit the migration of A549 cells.

In the cell scratch test, high, medium, and low concentrations of drugs 2,5-2Cl, 2Br-5Cl, 2-Cl, 2-F, and benzaldehyde were used for 6 h. Compared with the blank control group (0.9% DMSO), there was no statistical difference in the cell migration rate of each administration group. However, after 24 h treatment, the migration distances of 2,5-2Cl (1.25, 2.5 μmol/L), 2Br-5Cl (1.25, 2.5, 5 μmol/L), 2-Cl (1.25, 2.5, 5 μmol/L), and 2-F (5 μmol/L) in the drug group were significantly different from those in the control group. Compared with 0.9% DMSO, the migration distance was smaller (*p* < 0.01), with a very significant difference. The ability to inhibit the migration of A549 cells was sorted from strong to weak: 2,5-2Cl > 2Br-5Cl > 2-Cl > 2-F > benzaldehyde.

In addition, the preliminary experiments of this experiment revealed that the cells were starved in the basic medium, and their migration and invasion ability became poor. The migration area after 24 h of scratching was essentially the same as that at 0 h, and it could not be distinguished whether it was due to starvation or drug inhibition. However, the migration area and migration distance of the blank group were found to change significantly when complete medium was added for 24 h after the completion of the scratching operation. Therefore, complete medium was used for this experiment.

##### Effects of Five Promising Compounds on the A549 Cell Inhibition

Normal A549 cells grow into monolayer cells that attach to or cling to the culture flask, and when the cells are damaged or die, they are less able to adhere to the flask and, therefore, float on the liquid medium. The state of A549 cells after the action of five promising compounds at different concentrations was observed by inverted microscope. The cytotoxic effect of five promising compounds of piperidone monocarbonyl curcumin analogues on A549 cells was preliminarily demonstrated, which could promote cell death.

**Figure 6:**
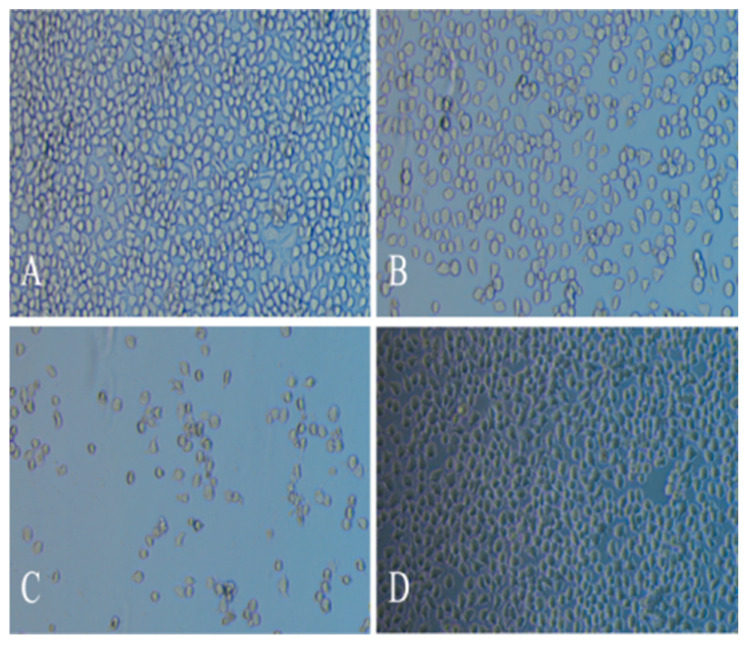
A549 cells treated with curcumin and 0.9% DMSO for 24 h (200×). (**A**) Curcumin 10 μmol/L group; (**B**) Curcumin 20 μmol/L group. (**C**) Curcumin 40 μmol/L group; and (**D**) 0.9% DMSO group.

**Figure 7:**
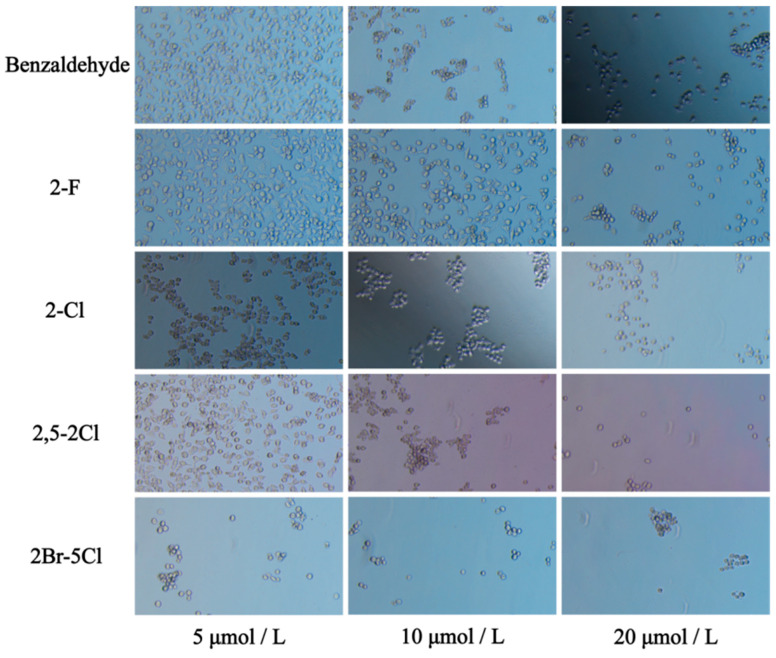
A549 cells treated with five promising piperidone monocarbonyl curcumin analogues for 24 h (200×).

Figure 6 and Figure 7 show the photos of A549 cells treated with curcumin and the five promising compounds at different concentrations for 24 h. The cells in the blank control group grew well and adhered closely to the wall. The morphological characteristics of apoptosis in different doses of piperidone monocarbonyl curcumin analogue groups were obvious; the cells floated, became loose, and the nucleus was concentrated into fragments. Partial experiment areas were left due to the blankness of cell death, which was particularly prominent in the high-dose drug group. Under the action of 2,5-2Cl, 2Br-5Cl, and 2-Cl at a low concentration of 5 μmol/L, most of the cells became round and loose, and the number of normal cells decreased significantly with the increase in drug concentration. The cytotoxic effects of the five promising compounds on A549 cells were dose-dependent. In addition, it can be seen from Figure 6 that the inhibitory effect of 2,5-2Cl, 2Br-5Cl, 2-Cl, 2-F, and benzaldehyde on A549 cells at the same concentration was significantly higher than that of curcumin, and the inhibitory effect of 2,5-2Cl was the strongest, which was also consistent with the results of the MTT assay.

##### Effects of Five Promising Compounds on Apoptosis of A549 Cells

In this study, we used a flow cytometer to detect the apoptosis rate of A549 cells treated with five promising compounds for 24 h. We found that these five promising compounds significantly promoted the early apoptosis of A549 cells and increased in a dose-dependent manner. The results suggest that the inhibition of tumour cell growth by these five promising compounds may be achieved through the induction of apoptosis.

**Figure 8:**
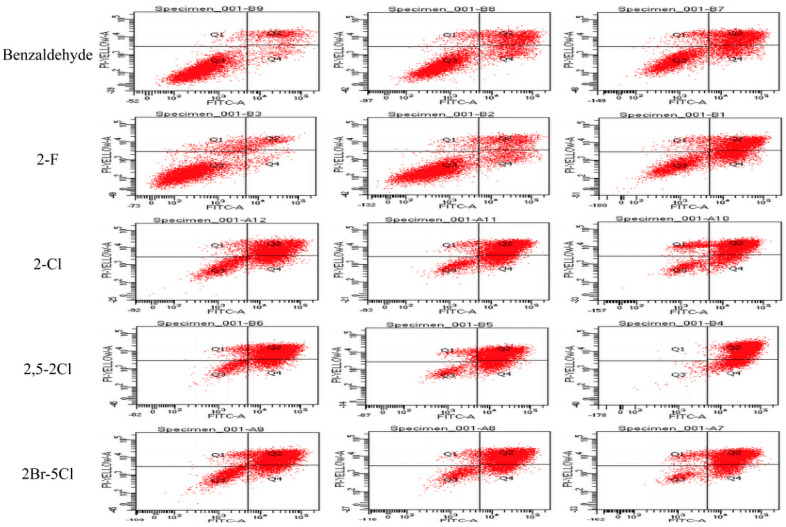
Flow chart of A549 cell apoptosis induced by five promising compounds.

**Figure 9:**
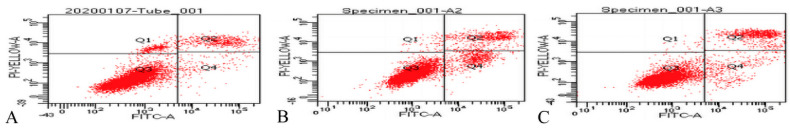
Flow chart of A549 cell apoptosis induced by 0.9% DMSO, curcumin, and cisplatin. (**A**) 0.9% DMSO; (**B**) Curcumin 20 μmol/L; (**C**) Cisplatin solution 30 μmol/L.

**Figure 10:**
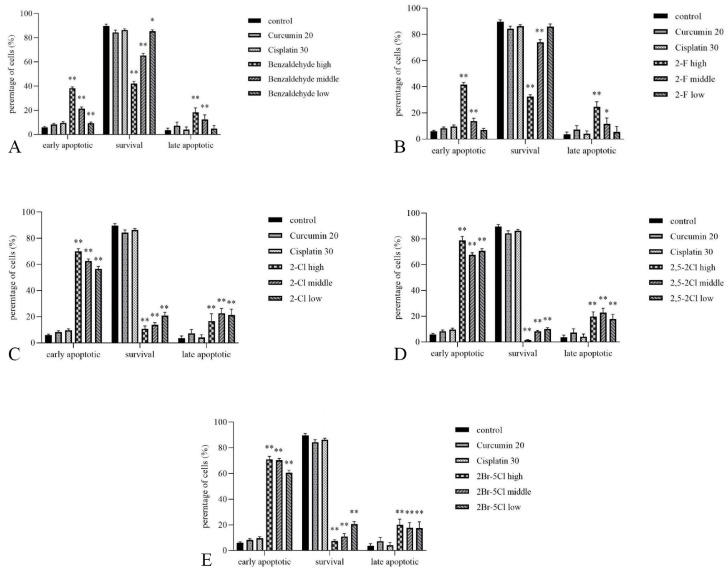
Bar graph of A549 cell apoptosis induced by five kinds of monocarbonyl curcumin. (**A**) 2,5-2Cl group; (**B**) 2Br-5Cl group; (**C**) 2-Cl group; (**D**) 2-F group; and (**E**) Benzaldehyde group. ***Note:*** Compared with blank control group: 0.9% DMSO, * p < 0.05; ** p < 0.01.

Flow cytometry results are shown in Figure 8 and Figure 9. The necrotic cell population appears in Q1 quadrant, the late-apoptotic cell population in Q2 quadrant, the normal cell population in Q3 quadrant, and the early-apoptotic cell population in Q4 quadrant. Statistical results are shown in Figure 10. As the drug dose increases, the proportion of normal cells decreases, and the proportion of early-apoptotic and late-apoptotic cells increases, which is positively correlated with the drug concentration, presenting a concentration-dependent relationship.

After A549 cells were treated with 20 μmol/L curcumin for 24 h, 84.27% of A549 cells were normal cells detected by flow cytometry, and after A549 cells were treated with 30 μmol/L cisplatin solution for 24 h, the proportion of normal cells was 86.27%, which was not statistically different from 89.53% of normal cells in the blank control group. Except for the low concentration of the 2-F drug group, the percentage of normal cells in other drug groups was significantly different from that in the blank control group (*p* < 0.01 or *p* < 0.05).

Among them, 2,5-2Cl had the strongest apoptosis-inducing effect on A549 cells, which was consistent with the IC_50_ results of piperidone monocarbonyl curcumin analogue on A549 cells determined by the MTT method, with the IC_50_ value of 2,5-2Cl on A549 cells being the smallest.

2,5-2Cl, 2Br-5Cl, 2-Cl, 2-F, benzaldehyde: High, medium, and low drug concentrations are 5 μmol/L, 2.5 μmol/L, and 1.25 μmol/L, respectively. Curcumin: 20 μmol/L, and cisplatin: 30 μmol/L.

##### Effects of Five Promising Compounds on the Expression Levels of Related Proteins in Apoptosis of A549 Cells

Apoptosis is the orderly cell death controlled by genes in order to maintain the stability of the internal environment. Apoptosis is a complex reaction process in many ways, and this experiment was conducted to explore the ways of apoptosis by measuring the expression levels of apoptosis-related proteins after the treatments of five promising compounds on A549 cells for 24 h.

**Figure 11:**
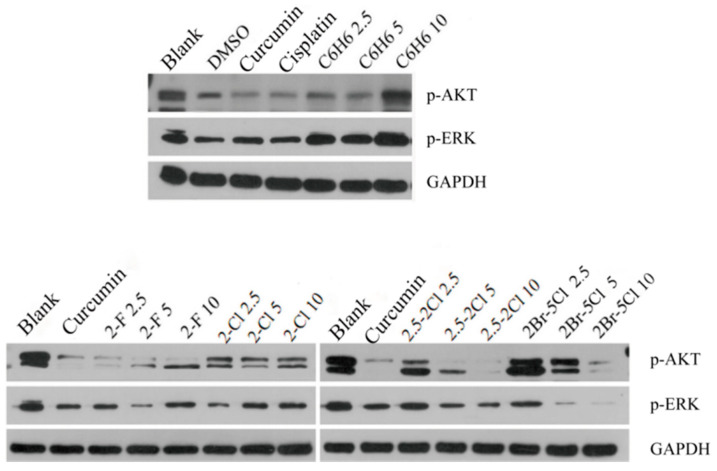
Protein band diagram.

**Figure 12:**
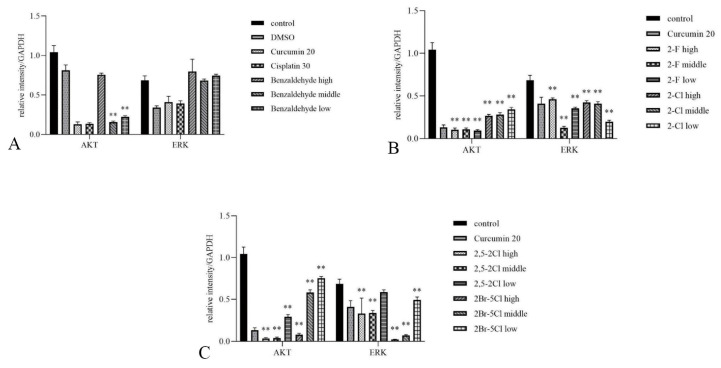
The statistical results of each group of protein bars. (**A**) Benzaldehyde group; (**B**) 2-F and 2-Cl groups; (**C**) 2,5-2Cl and 2Br-5Cl groups. ***Note:*** Compared with blank control group, * p < 0.05; ** p < 0.01.

In this experiment, A549 cells were treated with five promising compounds with a high concentration of 10 μmol/L, a medium concentration of 5 μmol/L, and a low concentration of 2.5 μmol/L for 24 h. The levels of AKT (protein kinase B) and ERK (extracellular regulated protein kinases) in cells were detected by Western blot. The results are shown in Figure 11. From the statistical results in Figure 12, it can be seen that, compared with the control group, the expression level of AKT protein in the five promising compound groups decreased significantly (*p* < 0.01 or *p* < 0.05), suggesting the inhibition of expression of AKT and induced apoptosis of A549 cells. Except for the benzaldehyde group, the ERK protein levels in the 2,5-2Cl, 2Br-5Cl, 2-F, and 2-Cl groups were significantly lower than those in the control group (*p* < 0.01 or *p* < 0.05), suggesting the inhibition of the expression of ERK in A549 cells and induced apoptosis.

**Figure 13:**
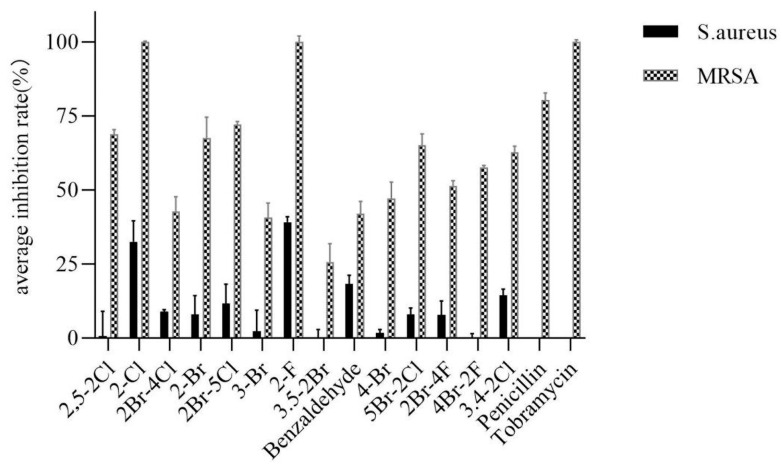
Anti-MRSA activity of fourteen curcumin analogues (160 μmol/L).

It is worth noting that 2,5-2Cl and 2Br-5Cl showed different degrees of inhibition on the expression of AKT and ERK. 2,5-2Cl mainly induced apoptosis by inhibiting the expression of AKT in A549 cells, while 2Br-5Cl mainly induced apoptosis by inhibiting the expression of ERK in A549 cells, as shown in Figure 13. With the increase in drug concentration, the inhibitory effect was significantly enhanced. In addition, compared with other promising compounds, 2,5-2Cl and 2BR-5Cl had stronger inhibitory effects on AKT and ERK protein expression, respectively.

2,5-2Cl, 2Br-5Cl, 2-Cl, 2-F, and benzaldehyde: High, medium, and low drug concentrations are 5 μmol/L, 2.5 μmol/L, and 1.25 μmol/L, respectively. Curcumin: 20 μmol/L, and cisplatin: 30 μmol/L.

#### 2.2.2. Antibacterial Activity of Curcumin Analogues on MRSA

To evaluate the antibacterial activity of the synthesized compounds against MRSA, the broth inhibition test and AGAR plate zone of inhibition test were used, and two first-line antibiotics, tobramycin and penicillin, were selected as positive control drugs.

##### Determination of Minimum Inhibitory Concentration (MIC) and Inhibition Curve of Promising Compounds Against MRSA

The results of the broth inhibition assays are shown in Figure 13. All compounds showed inhibition of MRSA at 160 μmol/L to different extents, with the promising compounds 2-Cl, 2-F, and the positive control drug tobramycin showing 100% inhibition of MRSA. Therefore, the inhibitory effects of 2-Cl, 2-F, and tobramycin on MRSA at low concentrations were detected. When the concentration of 2-Cl was 70 μmol/L, the inhibition rate was 100%, and the medium was clarified. When the concentration of 2-Cl was 60 μmol/L, the inhibition rate was less than 100% and the medium was cloudy. When the concentration of 2-F was 80 μmol/L, the inhibition rate was less than 100% and the medium was cloudy. When the concentration of tobramycin was 80 μmol/L, the inhibition rate was 100% and the medium was clear. When the concentration of tobramycin was 70 μmol/L, the inhibition rate was less than 100%, and the medium was cloudy. Therefore, the MIC of 2-Cl, 2-F, and tobramycin were 70, 160, and 80 μmol/L, respectively.

**Figure 14:**
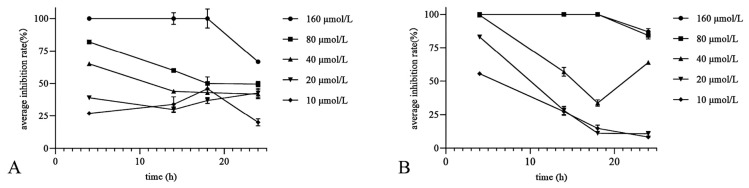
2-F and 2-Cl inhibition of MRSA curve. (**A**) 2-F group; (**B**) 2-Cl group.

In order to detect the trend of inhibition rate of the compounds with time, the dynamic inhibition rate of MRSA at different drug concentrations at different time points was examined, and the results are shown in Figure 14.

When the concentrations of 2-Cl were 160 μmol/L and 80 μmol/L, the inhibition rate was still 100% after 18 h, showing a strong antibacterial effect. After 24 h, the inhibition rate decreased to 87.02%, and the inhibition ability was weakened. At a low concentration of 2-Cl, the inhibition rate decreased rapidly from 4 to 18 h and tended to be stable from 24 to 28 h.

When the concentration of 2-F was 160 μmol/L, the inhibition rate was still 100% at 18 h, showing a strong antibacterial effect. After 24 h, the inhibition rate decreased to 66.64%, and the antibacterial ability was weakened. When the concentration of 2-F was 80 μmol/L, the inhibition rate was 82% after 4 h. The inhibition rate continued to decrease from 4 h to 18 h. The antibacterial capacity of 2-Cl and 2-F was concentration-dependent and weak at low concentrations.

##### Determination of Inhibition Zone of Promising Compounds

**Figure 15:**
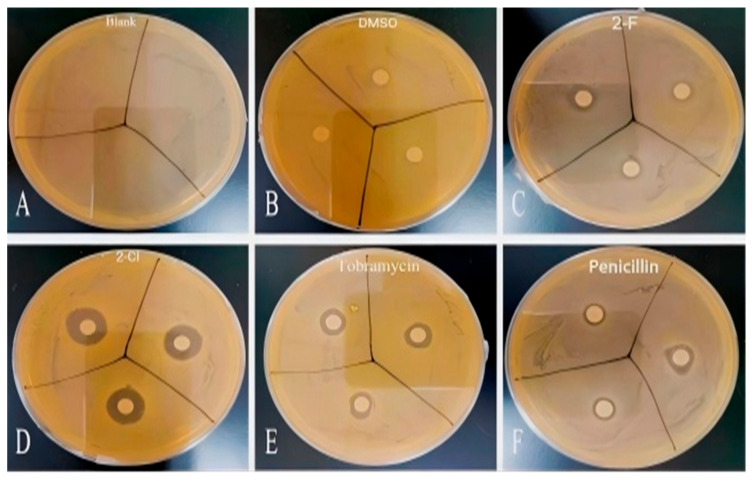
Inhibition zone of two kinds of monocarbonyl curcumin analogues on MRSA. (**A**) Blank group; (**B**) DMSO group; (**C**) 2-F group; (**D**) 2-Cl group; (**E**) Tobramycin group; and (**F**) Penicillin group.

The sizes of the antibacterial zones of the 2-F compounds 2-F and 2-Cl and the positive controls tobramycin, penicillin, blank, and DMSO against MRSA are shown in Figure 15, with the inhibition zones from largest to smallest: 2-Cl > tobramycin > 2-F > penicillin. The *p*-values for both the synthetic compound and positive drug groups were less than 0.01 compared to the control DMSO group, showing an extremely significant difference. The specific data are shown in Table 3.

From the above experiments, it can be seen that, among the 14 curcumin analogues synthesized, 2-Cl has the strongest antibacterial effect on MRSA, and its antibacterial activity is even higher than that of the positive drugs tobramycin and penicillin.

### 2.3. Results of Molecular Docking

It is generally believed that the binding energy less than −4.25 kcal/mol indicates that the ligand has a certain binding activity with the receptor, whereas, if its less than −5.0 kcal/mol, it suggests a better binding activity, and less than −7.0 kcal/mol has a strong binding activity [30]. The smaller value of binding energy indicates that there is a more stable binding conformation and a greater possibility of interaction between the target and the component. The results of molecular docking showed that the binding energy of the five promising compounds with the corresponding target protein AKT2 was less than −7.0 kcal/mol, suggesting that the five compounds had strong binding activity with AKT2, and better binding than the binding activity of curcumin with AKT2. The predicted binding strength of the five promising compounds with AKT2 was in the following order: 2,5-2Cl (−9.8 kcal/mol) > 2-Cl (−9.4 kcal/mol) > 2-F (−9.3 kcal/mol) > benzaldehyde (−9.1 kcal/mol) > 2Br-5Cl (−7.4 kcal/mol) > curcumin (−6.8 kcal/mol). Except for the fact that that 2-Cl had no binding affinity with ERK1, the binding energies of the other four promising compounds with the corresponding target protein ERK1 were all less than −7.0 kcal/mol, suggesting that the four compounds had strong binding activity with ERK1 and were superior to the binding activity of curcumin with ERK1. The binding strength of ERK1 was in the following order: 2-F (−10.2 kcal/mol) > benzaldehyde (−9.7 kcal/mol) > 2Br-5Cl (−9.4 kcal/mol) > 2. 5-2Cl (−9.3 kcal/mol) > curcumin (−7.9 kcal/mol) > 2-Cl (−0.0 kcal/mol). The minimum binding energies of the five compounds to AKT2 and ERK1 are shown in Supplementary Materials.

**Figure 16:**
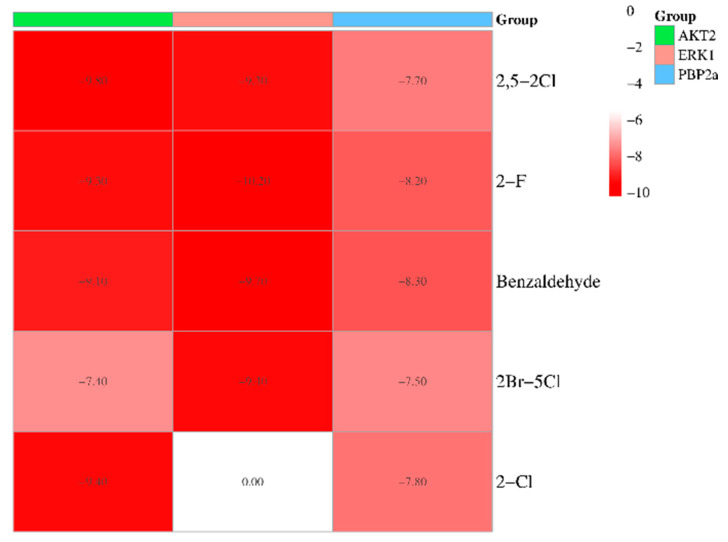
Heatmap of the binding energy of the five promising compounds with AKT2, ERK1, and PBP2a.

The binding energies of the five promising compounds with the corresponding target protein PBP2a of MRSA were all less than −7.0 kcal/mol, suggesting that the five compounds had strong binding activity with PBP2a, and were superior to the binding activity of penicillide, curcumin, and tobramycin with PBP2a. The binding degree of the five promising compounds with PBP2a was in the following order: Benzaldehyde (−8.3 kcal/mol) > 2-F (−8.2 kcal/mol) > 2-Cl (−7.8 kcal/mol) > 2,5-2Cl (−7.7 kcal/mol) > 2Br-5Cl (−7.5 kcal/mol) > penicillide (−7.2 kcal/mol) > curcumin (−6.6 kcal/mol) > tobramycin (−6.3 kcal/mol). The minimum binding energies of the five compounds to PBP2a are shown in Supplementary Materials. The binding energy (kcal/mol) heatmap of the five promising compounds with AKT2, ERK1, and PBP2a are shown in Figure 16. The more red the colour order, the stronger the binding.

The binding mode of the five compounds to AKT2 was mainly a hydrophobic interaction. For example, with the strongest binding affinity to AKT2, compound 2,5-2Cl was observed in a hydrophobic interaction with amino acid residues LEU158, PHE163, VAL166, and PHE439 of AKT2, respectively. In addition, in addition to hydrophobic interaction, 2-Cl and 2Br-5Cl also have a halogen bond with the amino acid residues LYS160 or ASN325 of AKT2, respectively, and the compound benzaldehyde has a hydrogen bond with the amino acid residue GLU279 of AKT2. The binding mode of the four compounds to ERK1 was also dominated by a hydrophobic interaction. For example, a hydrophobic interaction was observed between the amino acid residues ILE48, TYR53, VAL56, LYS71, ILE73, LEU124, LEU173, and ASP184 of ERK1 and the compound 2-F, with the strongest binding affinity to ERK1. In addition, there is a π-Cation interaction with residue LYS71, and the force of benzaldehyde and 2-F on ERK1 is similar. The other promising compounds only have hydrophobic interactions with ERK1.

The five compounds bind to PBP2a mainly by hydrophobic interaction. For example, there is a hydrophobic interaction between compound 2-Cl and amino acid residues TYR272, LYS273, ALA276, LYS289, GLN292, and GLU294 of PBP2a, which is more than other compounds. Compound 2-F not only has a hydrophobic interaction with amino acid residues ARG151, THR165, and TYR373 of PBP2a, but also has a hydrogen bond connection with amino acid residue ARG241. Compound benzaldehyde has a hydrophobic interaction with amino acid residues TYR272, LYS273, ALA276, LYS289, GLN292, and GLU294 of PBP2a, and amino acid residues LYS273 and HIS293 are connected by a hydrogen bond, and there is also a π-Cation interaction with amino acid residue LYS289 of PBP2a.

**Figure 17:**
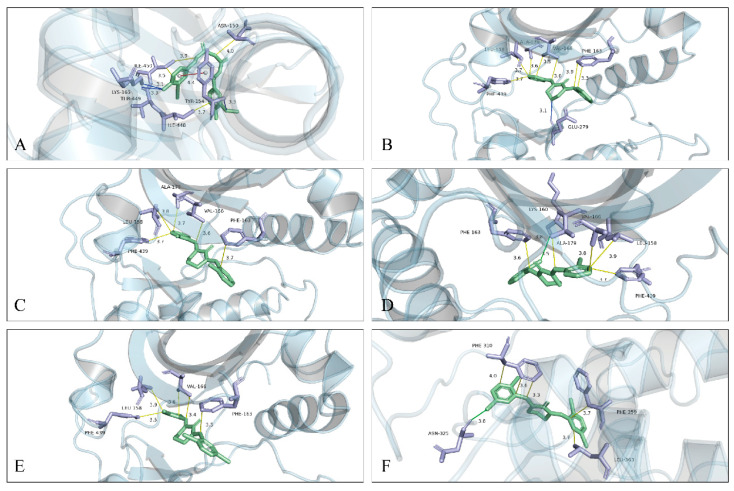
Visualization diagram of the molecular docking of the five promising compounds with AKT2. (**A**) Curcumin group; (**B**) Benzaldehyde group; (**C**) 2-F group; (**D**) 2-Cl group; (**E**) 2,5-2Cl group; and (**F**) 2Br-5Cl group.

**Figure 18:**
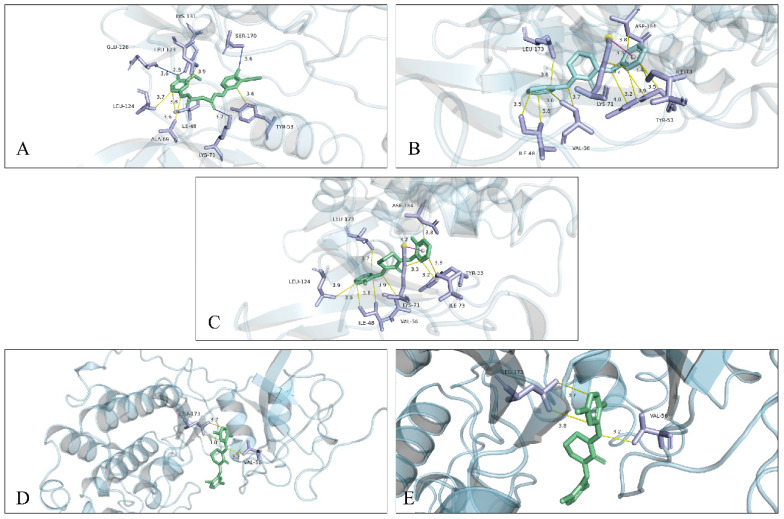
Visualization diagram of the molecular docking of the five promising compounds with ERK1. (**A**) Curcumin group; (**B**) Benzaldehyde group; (**C**) 2-F group; (**D**) 2,5-2Cl group; and (**E**) 2Br-5Cl group.

**Figure 19:**
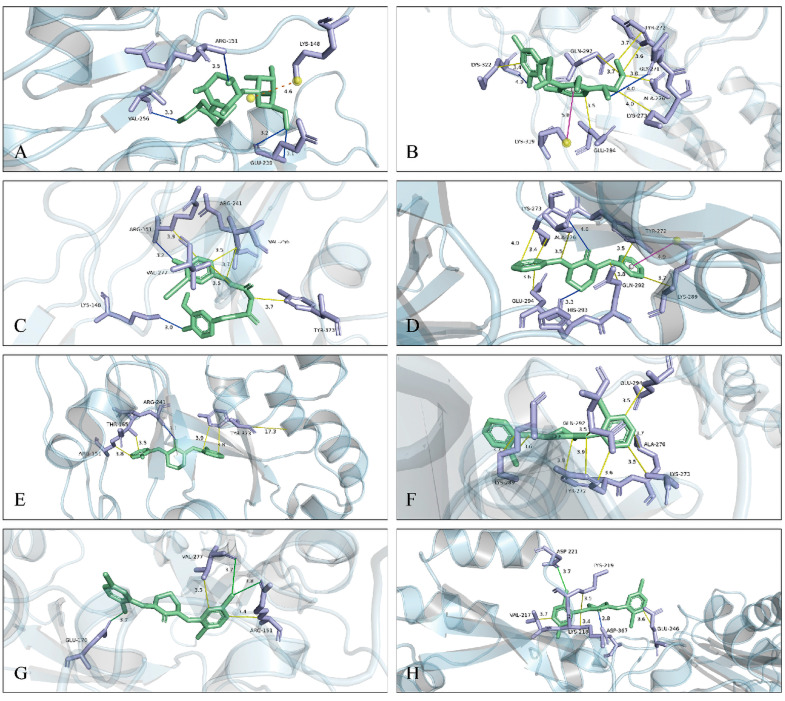
Visualization diagram of the molecular docking of the five promising compounds with PBP2a. (**A**) Tobramycin group; (**B**) Penicillin group; (**C**) Curcumin group; (**D**) Benzaldehyde group; (**E**) 2-F group; (**F**) 2-Cl group; (**G**) 2,5-2Cl group; and (**H**) 2Br-5Cl group.

A hydrophobic interaction enhances the stability of the compound structure. The spatial structure after binding is relatively stable, and the docking state is good. The visualization maps of molecular docking of the five promising compounds with AKT2, ERK1, and PBP2a are shown in Figure 17, Figure 18 and Figure 19, respectively. The interaction types of the five promising compounds with AKT2, ERK1, and PBP2a are shown in Supplementary Materials, respectively.

### 2.4. ADMET Prediction

The prediction results are shown in Supplementary Materials. Except for 2Br-5Cl, the bioavailability scores of the other four promising compounds are higher. Synthetic accessibility score from 1 to 10 is from very easy to very difficult. The five promising compounds are easy to synthesize, and the difficulty of synthesis is lower than curcumin, penicillide, and tobramycin. Compared with compounds 2,5-2Cl and 2Br-5Cl, the other three promising compounds all conform to the Lipinski rule [31] ((1) molecular weight is less than 500; (2) the number of hydrogen bond donors is less than 5; (3) the number of hydrogen bond acceptors is less than 10; (4) the lipid–water partition coefficient is less than 5; and (5) the number of rotatable bonds is not more than 10.), and, therefore, have drug-like properties. In terms of absorption, 2,5-2Cl and 2Br-5Cl had a large lipid–water partition coefficient, LogP > 5, and strong lipophilicity affected drug absorption. The five promising compounds all have high gastrointestinal absorption and are not substrates of P-glycoprotein, and the predicted drug resistance is not strong [32,33]. In terms of distribution, the five promising compounds can pass through the blood–brain barrier and bind more to plasma proteins; most of them are invalid binding states, there are few free drugs in the body, and the transport is stable. In terms of metabolism, the five promising compounds do not inhibit all CYP (cytochrome P450 proteins) enzymes, reducing the potential reaction with other drugs. In addition, the P450 enzyme is an important enzyme in liver metabolism. The main enzymes responsible for drug metabolism are 2D6 and 3A4 enzymes in the P450 enzyme family. No promising compound is an inhibitor of both enzymes, reducing the risk of drug accumulation and poisoning [34]. In terms of excretion, a total clearance rate below 2.0 is considered to be lower [35]. The total clearance rate of the five promising compounds is lower than 1. The compounds have high stability in the body and will not be rapidly excreted. It can persist in the body for a long time and may achieve the expected therapeutic goals. Organic cation transporter 2 (OCT2) is the most abundant and important efflux transporter involved in renal excretion of cationic drugs [36]. Whether it is an OCT2 substrate not only affects its clearance rate but also suggests that it affects the possibility of adverse reactions caused by affecting other drug clearance rates [37]. Except for benzaldehyde, the other four promising compounds were OCT2 substrates. In terms of toxicity, none of the five promising compounds in the Ames mutation prediction were mutagenic and carcinogenic [34], but the risk of causing hepatotoxicity and nephrotoxicity was predicted, which is consistent with the excretion. Oral rat acute toxicity (LD50) was higher than that of curcumin, and double-substituted compounds were higher than single-substituted compounds.

### 2.5. Discussion

In this study, a series of piperidone monocarbonyl curcumin analogues were synthesized according to the previous structural studies of curcumin analogues, half of which were new compounds. Their anti-tumour activities against common cancers were evaluated, including human hepatocellular carcinoma cell line (HepG2), human cervical cancer cell line (HeLa), human breast cancer cell line (MCF-7), and human non-small cell lung cancer cell line (A549). It is worth noting that five curcumin analogues, benzaldehyde, 2-F, 2-Cl, 2,5-2Cl, and 2Br-5Cl, have shown excellent inhibitory effects on four cancer cell lines, of which 2,5-2Cl and 2Br-5Cl are new compounds. The five promising compounds were most sensitive to the A549 cell line, with IC_50_ below 5 μmol/L.

In the MTT assay, 2-F and benzaldehyde had a stronger inhibitory effect on the proliferation of the A549 cell line. However, the pharmacodynamic and time-effect results obtained from the CCK-8 experiment showed that the rapid inhibition intensity of 2-F and benzaldehyde was low, and the inhibition of the proliferation of A549 cells was weak at low concentrations. The results of apoptosis of A549 cells detected by flow cytometry can also evaluate the overall ability of the five promising compounds to induce apoptosis of A549 cells from strong to weak: 2,5-2Cl > 2Br-5Cl > 2-Cl > 2-F (EF24) > benzaldehyde.

In view of the anti-tumour activity of curcumin analogues, we draw the following conclusions: (1) The overall biological activity of 2-chloro-substituted analogues and 2-fluoro-substituted analogues on the benzene ring is better than that of unsubstituted analogues on the benzene ring. (2) The biological activity of 2-and 5-disubstituted analogues substituted by chlorine is better than that of 2-bromo- and 5-chloro-substituted analogues. The overall biological activity of 2-chloro-substituted analogues on the benzene ring is higher than that of 2-fluoro-substituted analogues on the benzene ring, and the activity of 2-fluoro-substituted analogues is higher than that of 2-bromo-substituted analogues on the benzene ring. (3) The biological activity of chlorine-substituted 2-position- and 5-position-disubstituted analogues is better than that of chlorine-substituted 3-position- and 4-position-disubstituted analogues. The biological activity of 2-bromo-substituted and 5-chloro-substituted disubstituted analogues is better than that of 2-bromo-substituted and 4-chloro-substituted disubstituted analogues. (4) The anti-A549 cell activity of 2- and 5-disubstituted analogues substituted by chlorine was higher than that of 2- and 5-disubstituted analogues substituted by bromine. When the substitution positions of the halogens of the 2-bromo-substituted and 5-chloro-substituted disubstituted analogues were exchanged to the 2-chloro-substituted and 5-bromo-substituted disubstituted analogues, the biological activity was reduced, and the activity against A549 cells was significantly reduced by about three times.

The structure–activity relationship (SAR) between the structure and anti-tumour activity of piperidone monocarbonyl curcumin analogues was analyzed, and the relationship between the halogen substitution position on the benzene ring and the anti-A549 cell line activity was speculated: (1) The introduction of halogen substitution is a common strategy to improve the biological activity of compounds. Whether it is the difference in halogen atoms or the change in substituent position, it will affect the change in electron cloud density of compounds, thus affecting the pharmacological effects of compounds; (2) at the ortho position (2 position) of the benzene ring, the strong electron-withdrawing substituent is beneficial to enhance the cytotoxic activity of the analogues [38]; the overall biological activity of chlorine-substituted analogues is better than that of bromine-substituted or fluorine-substituted compounds. It is speculated that chlorine atoms may be related to the apoptosis of lung cancer tumour cells; (3) the biological activity of para-halogen-substituted analogues on both sides of the benzene ring of piperidone monocarbonyl curcumin analogues is higher than that of ortho- or meta-substituted analogues on the benzene ring; and (4) at the 5-position, strong electron-withdrawing substituents are beneficial to enhance the biological activity of the compound, and the effect of 5-position substitution on biological activity is greater than that of other positions.

As an inhibitor of receptor tyrosine kinases, curcumin can target a large number of signalling molecules alone, such as MAPK, PI3K/Akt, JAK/STAT, and NF-κB pathways, which are involved in basic cellular processes such as proliferation, apoptosis, and migration [39]. Among them, Akt (protein kinase B) and ERK (extracellular regulated protein kinases) in the MAPK pathway are two major cell survival pathways that are upregulated and activated in lung cancer tissues [40]. It is worth noting that there is a compensatory feedback loop between the two signalling pathways in lung cells: Inhibition of AKT induces ERK activation, whereas inhibition of ERK leads to AKT activation. Compensatory activation of these key intracellular signalling pathways can lead to limited anti-tumour activity and drug resistance of a single inhibitor [41].

Under Western blot detection, except for benzaldehyde, the other promising compounds showed the ability to inhibit the expression of AKT and ERK in A549 cells to varying degrees. Curcumin analogues exert anti-lung cancer function through multiple targets and can also be used together with other drugs to enhance anti-lung cancer activity and reduce adverse reactions. Some scholars have found that the use of chemical inhibitors to block any one of the proteins will moderately sensitize cisplatin-induced apoptosis in A549 cells and reduce cell viability, while simultaneous inhibition of p-ERK1/2 and p-Akt can significantly enhance the cytotoxicity of cisplatin [41]. In general, the inhibitory effect of the five promising compounds on the expression of AKT protein was stronger than that of ERK protein. The Swiss target prediction was used to predict the five promising compounds, which also showed that the first and third most possible active sites were AKT2 and AKT1, respectively. The down-regulation of AKT protein expression by 2,5-2Cl was the most obvious, while the down-regulation of ERK protein expression by 2Br-5Cl was the most obvious, indicating that 2,5-2Cl and 2Br-5Cl mainly regulate and induce cell death through different pathways.

Penicillin-binding proteins (PBPs) are one of the molecules that play an important role in the synthesis of peptidoglycan, thereby forming bacterial cell walls. β-lactam antibiotics can inhibit the growth of methicillin-resistant *Staphylococcus aureus* (MRSA) by targeting the transpeptidase on PBPs. However, penicillin-binding protein 2a (PBP2a) has a low affinity for β-lactam penicillin and antibiotics, which can replace the function of PBP, resulting in the existence of the MRSA resistance mechanism to antibiotics. Therefore, PBP2a is a common potential target for inhibiting the growth of MRSA [42]. Molecular docking prediction showed that the binding affinity of 2-F and 2-Cl to PBP2a was stronger than that of positive drugs tobramycin and penicillin, suggesting that 2-Cl had better anti-MRSA effect than antibiotics in vitro, and 2-F also had better anti-MRSA effect. A series of piperidone monocarbonyl curcumin analogues has a certain inhibitory effect on MRSA. The MIC of compound 2-Cl was lower than tobramycin and penicillin, and the inhibitory effect of 2-F on MRSA was stronger than penicillin.

It is worth noting that the ADMET prediction results show that all five promising compounds can pass through the blood–brain barrier, suggesting that curcumin analogues have the potential to develop a treatment for brain diseases. Studies have found that monocarbonyl curcumin analogues have neuroprotective potential and can improve cognitive and memory abilities [43]. Curcumin analogues have the ability to reduce oxidative stress and work as antidepressants [44].

In addition, although the IC_50_ values of the five promising compounds to human normal liver cells (L02) were much higher than their toxicity to tumour cells, there was no mutagenicity in the prediction of Ames mutation, and the LD_50_ values of the five promising compounds in the predicted oral rat acute toxicity were greater than those of tobramycin and curcumin, but the risk of hepatotoxicity and nephrotoxicity was predicted, which needed to be further verified at the animal level.

In general, 2-Cl and 2-F have the potential to develop into better anti-tumour and antibacterial dual bioactive compounds, which are in line with the Lipinski rule and have better druggability. 2,5-2Cl and 2Br-5Cl have the strongest inhibitory effect on A549 lung cancer cells, but their lipid–water partition coefficients are larger, and the molecular weight of 2Br-5Cl is slightly larger than 500, which needs further improvement and development of pharmaceutical content. The bioavailability of the five promising compounds needs to be improved. Most of the curcumin drug delivery systems currently developed still have a lot of room for improvement in targeting selectivity to specific tumour tissues, improving drug utilization at the site of action, and producing higher activity and fewer adverse reactions. In the future, the development of a structure–activity relationship, potential molecular mechanism, novel multiple bioactive analogues, and drug delivery system of curcumin and its analogues will be the focus of our continuous research.

## 3. Materials and Methods

### 3.1. Experimental Materials and Instruments

All reagents and solvents were purchased from commercial suppliers and can be used without further purification. The 4-piperidone hydrochloride hydrate came from the following manufacturer: Shanghai Yuanye Biological, China; the 2-fluorobenzaldehyde, 2-chlorobenzaldehyde, 3,5-dibromobenzaldehyde, 2-bromobenzaldehyde, 4-bromo-2-fluorobenzaldehyde, 2-bromo-5-chlorobenzaldehyde, 5-bromo-2-chlorobenzaldehyde, 4-bromo-benzaldehyde, 3-bromo-benzaldehyde, 2-bromo-4-fluorobenzaldehyde, 2-bromo-4-chlorobenzaldehyde, 3,4-dichlorobenzaldehyde, and 2,5-dichlorobenzaldehyde all came from Shanghai Macklin Biochemical Technology Co., Shanghai, China); Methanol (Manufacturer: Sigma-Aldrich, St. Louis, MO, USA); Sodium hydroxide (Manufacturer: Beijing Chemical Plant, Beijing China); and benzaldehyde (Manufacturer: Fuchen Chemical Reagent Co., Ltd., Tianjin, China).

ZNCL-BS intelligent magnetic agitator came from Gongyi Yuhua Instrument Co., Ltd., Gongyi, China; Agilent Technologies’ 6520 Accurate-Mass Q-TOF LC/MS came from Agilent Technologies, Inc., Santa Clara, CA, USA; BT125D Electronic analysis balance came from Beijing Sartorius Instrument System Engineering Co., Ltd., Beijing, China; AM-500 NMR instrument came from Bruker Corporation, Zurich Switzerland; Waters UPLC: Binary solvent management system, on-line degasifier, automatic sampler, and PDA detector came from Waters Corporation, MA USA; ultrasonic cleaner came from Jiangsu Kunshan Ultrasonic Instrument Co., Ltd., Kunshan, China; 101-1AB Electric blast drying oven came from Tianjin Sairis Experimental Analysis Instrument Factory, Tianjin, China.

#### 3.1.1. Synthesis of Monocarbonyl Curcumin Analogues of Piperidones [45]

The molar mass ratio of the reactant 4-piperidone hydrochloride to substituted benzaldehyde was 1:2. After dissolving 4-piperidone hydrochloride in ethanol, 2 molar mass of substituted benzaldehyde was added. The reaction catalyst was 20% aqueous sodium hydroxide, and the temperature of the reaction system was room temperature. The reaction was closely monitored by silica gel thin-layer chromatography (TLC) and a yellow or light-yellow precipitate was formed after 1.5–2 h.

A total of 1 mmol of 4-piperidone hydrochloride was dissolved in 6 mL of methanol, placed on a magnetic stirrer, and placed in a rotor (500 r/min). A total of 2 mmol of substituted benzaldehyde was added drop by drop, and then 1.5 mL of 20% sodium hydroxide aqueous solution was slowly added drop by drop to the reaction system. The reaction was stirred at room temperature, and the reaction process was tracked by TLC. After 2 h of reaction, 1 mL of 12 mol of hydrochloric acid (12 mol/L) was quickly added, and the reaction continued for 20 min. After standing for 30 min, the filter residue was washed with methanol several times after filtration, and the purity was detected by ultra-high performance liquid chromatography. The detection conditions were as follows: mobile phase: 0.5% formic acid aqueous solution was aqueous phase, acetonitrile was organic phase; gradient elution conditions were 0–20 min, aqueous phase, 10–90%. Purified samples were dried at 37 °C in an oven to constant weight.

##### Synthesis of 3,5-Dibenzylidene-4-piperidone (Benzaldehyde/C6H6)

At 4.826 min and under 337.5 nm, a strong absorption peak area accounted for more than 97% of the total peak area. Light yellow powder, yield 45.61%. ^1^H-NMR (400 MHz, DMSO): δ (ppm) 9.98 (s, 1H), 7.90 (d, 2H), 7.55 (m, 10H), and 4.49 (s, 4H). ^13^C-NMR (100 MHz, CDCl3): δ (ppm) 44.29 (C-11, C-12), 129.43 (C-13, C-14, C-17, C-20), 128.41 (C-7, C-16), 130.54 (C-6, C-8, C-15, C-19), 131.01 (C-3, C-9), 134.18 (C-2, C-10), 139.61 (C-4, C-5), and 182.90 (C-18). HRMS (ESI) *m*/*z*: 276.1387 [M+H]+, calcd. for C_19_H_17_, NO 276.1387. ChemDraw 20.0-predicted melting point: 561.82.

##### Synthesis of 3,5-Bis(2-fluoro benzyl)-4-piperidinone (2-F)

A strong absorption peak was observed at 6.109 min under 320 nm. There was a strong absorption peak at 6.109 min at 250 nm, and a weak absorption peak at 5.290 min. Filtrate the reaction liquid to achieve a light-yellow residue. A small amount of filter residue was dissolved in methanol, filtered through a microporous membrane, and tested in the liquid phase. There was only one strong absorption peak at 320 nm and 250 nm at 6.109 min, and the peak area accounted for more than 99% of the total peak area. Light yellow powder yield was 75%. ^1^H-NMR (400 MHz, DMSO): δ (ppm) 8.34 (s, 1H), 7.79 (s, 2H), 7.60–7.52 (t, 2H), 7.52–7.45 (t, 2H), 7.40–7.14 (dd, J = 8.3, 7.3 Hz, 4H), and 4.16 (s, 4H). ^13^C-NMR (100 MHz, CDCl3): δ (ppm) 45.77 (C-22), 45.79 (C-16), 116.25 (C-19), 116.47 (C-9), 122.46 (C-12), 122.33 (C-14), 125.20 (C-7), 125.23 (C-2), 129.50 (C-17), 129.55 (C-20), 131.46 (C-1), 131.48 (C-4), 132.42 (C-21), 132.50 (C-17), 133.75 (C-13, C-23), 159.55 (C-18), 162.03 (C-6), and 184.89 (C-20). HRMS (ESI) *m*/*z*: 312.1219 [M+H]+, calcd. for C_19_H_15_F_2_, NO 312.1219. ChemDraw-predicted melting point: 588.04.

δ 8.33 (s, 1H), 7.79 (d, J = 2.0 Hz, 10H), 7.61–7.45 (m, 22H), 7.37 (d, J = 9.5 Hz, 12H), 7.33 (dd, J = 7.3, 1.3 Hz, 11H), 7.30–7.14 (m, 4H), and 4.16 (d, J = 2.1 Hz, 19H).

##### Synthesis of 3,5-Bis(2-chloro-benzyl)-4-piperidinone (2-Cl)

A strong absorption peak was observed at 6.297 min under 320 nm. There was a strong absorption peak at 6.289 min at 250 nm, and a weak absorption peak at 5.090 min. Filtrate the reaction liquid to achieve a light-yellow residue. A small amount of filter residue was dissolved in methanol, filtered through a microporous membrane, and tested in the liquid phase. There was only one strong absorption peak at 320 nm and 250 nm at 6.280 min, and the peak area accounted for more than 99% of the total peak area. Light yellow powder yield of 80%. ^1^H-NMR (400 MHz, DMSO): δ (ppm) 9.99 (s, 1H), 8.00 (s, 2H), 7.67–7.66 (d, J = 7.8 Hz, 2H), 7.65–7.62 (d, J = 7.6 Hz, 2H), 7.57–7.53 (dd, J = 7.5, 7.3 Hz, 2H), 7.52–7.47 (dd, J = 7.4, 7.3 Hz, 2H), and 4.36 (s, 4H). ^13^C-NMR (100 MHz, CDCl3): δ (ppm) 44.11 (C-3, C-6), 126.01 (C-14, C-22), 128.40 (C-15, C-23), 130.17 (C-11, C-21), 131.26 (C-13, C-16), 132.08 (C-12, C-17), 132.18 (C-9, C-10), 134.55 (C-2, C-7), 136.33 (C-8, C-1), and 182.69 (C-5). HRMS (ESI) *m*/*z*: 344.0602 [M+H]+, calcd. for C_19_H_15_Cl_2_, NO 344.0602. ChemDraw-predicted melting point: 646.70.

1H NMR (400 MHz, DMSO-d6) δ 9.99 (s, 1H), 8.00 (d, J = 1.9 Hz, 2H), 7.79–7.57 (m, 2H), 7.57–7.36 (m, 6H), and 4.36 (d, J = 2.2 Hz, 3H).

##### Synthesis of 3,5-Bis(2-bromo-benzyl)-4-piperidone (2-Br, New Derivative)

A strong absorption peak was observed at 6.608 min under 320 nm. At 250 nm, there was a strong absorption peak at 6.208 min and a weak absorption peak at 5.626 min. Filtrate the reaction liquid to achieve a light-yellow residue. A small amount of filter residue was dissolved in methanol, filtered through a microporous membrane, and tested in liquid phase. There was only one strong absorption peak at 320 nm and 250 nm at 6.770 min, and the peak area accounted for more than 99% of the total peak area. Light yellow powder yield of 78%. ^1^H-NMR (400 MHz, DMSO): δ (ppm) 10.34 (s, 1H), 7.92 (s, 2H), 7.90–7.86 (d, J = 7.8 Hz, 2H), 7.85–7.83 (d, J = 7.6 Hz, 2H), 7.82–7.56 (d, J = 7.8 Hz, 2H), 7.65–7.40 (m, 2H), and 4.32 (s, 4H). ^13^C-NMR (100 MHz, CDCl3): δ (ppm), 43.84 (C-6, C-16), 125.09 (C-5, C-21), 128.50 (C-4, C-9), 129.87 (C-3, C-17), 130.58 (C-11, C-12), 131.25 (C-2, C-20), 132.12 (C-10, C-18), 133.95 (C-1, C-14), 138.59 (C-8, C-15), and 182.72 (C-7). HRMS (ESI), *m*/*z*: [M+H]+, calcd. For C_19_H_15_Br_2_, NO 433.9562. ChemDraw-predicted melting point: 706.46.

##### Synthesis of 3,5-Bis(3-bromo-benzyl)-4-piperidone (3-Br, New Derivative)

At 7.685 min under 328 nm, a strong absorption peak area accounted for more than 91.3% of the total peak area. There was a light-yellow powder yield of 53.41%. ^1^H-NMR (400 MHz, DMSO): δ (ppm) 10.38 (s, 1H), 10.02 (s, 2H), 7.90–7.86 (d, J = 7.8 Hz, 2H), 7.73–7.65 (d, J = 7.8 Hz, 2H), 7.22–7.45 (m, 2H), 4.44 (s, 2H), and 3.21 (s, 4H). ^13^C-NMR (100MHz, CDCl3): δ (ppm) 43.91 (C-2, C-7), 122.60 (C-9, C-10), 129.69 (C-22, C-23, C-1, C-5), 131.42 (C17, C-19), 133.04 (C-20, C-12), 133.20 (C-13, C-15), 136.58 (C-4, C-11), 138.01 (C-3, C-18), and 182.72 (C-8). HRMS (ESI) *m*/*z*: 433.9574 [M+H]+, calcd. for C_19_H_15_Br_2,_ NO 433.9574. ChemDraw-predicted melting point: 706.46.

##### Synthesis of 3,5-Bis(4-bromo-benzyl)-4-piperidinone (4-Br, New Derivative)

Under 340 nm, there was a strong absorption at 7.508 min, and the peak area accounted for more than 95% of the total peak area. There was a light-yellow powder yield of 79.19%. ^1^H-NMR (400 MHz, DMSO): δ (ppm) 10.44 (s, 1H), 7.90–7.77 (d, J = 8.5 Hz, 4H), 7.60–7.46 (d, J = 8.5 Hz, 4H), 4.42 (s, 2H), and 3.21 (s, 4H). ^13^C-NMR (100 MHz, CDCl3): δ (ppm) 43.97 (C-3, C-19), 124.08 (C-11, C-16), 132.38 (C-8, C-9, C-12, C-15), 132.91 (C-1, C-2, C-7, C-10), 133.39 (C-17, C-18), 138.30 (C-4,C-5), and 182.72 (C-21). HRMS (ESI) *m*/*z*: 433.9562 [M+H]+, calcd. for C_19_H_15_Br_2_, NO 433.9562. ChemDraw-predicted melting point: 706.46.

##### Synthesis of 3,5-Bis(2,5-dichlorobenzyl)-4-piperidone (2,5-2Cl, New Derivative)

At 8.389 min under 305 nm, a strong absorption peak area accounted for more than 93% of the total peak area. There was a light-yellow powder yield of 50.45%. ^1^H-NMR (400 MHz, DMSO): δ (ppm) 10.10 (s, 1H), 7.87 (s, 2H), 7.68 (d, J = 8.6 Hz, 2H), 7.64 (d, J = 2.5 Hz, 2H), 7.60 (dd, J = 8.6, 2.5 Hz, 2H), and 4.38 (s, 4H). ^13^C-NMR (100 MHz, CDCl3): δ (ppm) 43.87 (C-7, C-14), 130.42 (C-12, C-21), 131.12 (C-10, C-17), 131.58 (C-1, C-18), 131.96 (C-15, C-20), 132.50 (C-11, C-24), 133.06 (C-5, C-9), 134.07 (C-2, C-3), 135.28 (C-4, C-8), and 182.48 (C-13). HRMS (ESI) *m*/*z*: 413.9802 [M+H]+, calcd. for C_19_H_13_Cl_4_, NO 413.9802. ChemDraw-predicted melting point: 731.58.

##### Synthesis of 3,5-Bis(3,4-dichlorobenzylidene)-4-piperidone (3,4-2Cl, New Derivative)

At 8.781 min under 334.4 nm, a strong absorption, the peak area accounted for more than 97% of the total peak area. There was a light-yellow powder yield of 51.82%. ^1^H-NMR (400 MHz, DMSO): δ (ppm) 10.18 (s, 1H), 7.87 (s, 2H), 7.84–7.75 (m, 4H), 7.53 (dd, J = 8.4, 2.1 Hz, 2H), and 4.47 (s, 4H). ^13^C-NMR (100 MHz, CDCl3): δ (ppm) 44.00 (C-1, C-6), 129.93 (C-17, C-18), 130.80 (C-5, C-25), 131.45 (C-10, C-12), 132.15 (C-18, C-19), 132.59 (C-20, C-24), 133.03 (C-3, C-11), 134.82 (C-7, C-8), 137.23 (C-2, C-13), and 182.55 (C-9). HRMS (ESI) *m*/*z*: 413.9808 [M+H]+, calcd. for C_19_H_13_Cl_4_, NO 413.9808. ChemDraw-predicted melting point: 731.58.

##### Synthesis of 3,5-Bis(2-bromo-4-fluorobenzyl)-4-piperidone (2Br-4F, New Derivative)

At 7.873 min under 323.3 nm, a strong absorption peak area accounted for more than 96.5% of the total peak area. There was a light-yellow powder yield of 82.24%. ^1^H-NMR (400 MHz, DMSO): δ (ppm) 10.38 (s, 1H), 7.86 (s, 2H), 7.81 (dd, J = 8.5, 2.6 Hz, 2H), 7.57 (dd, J = 8.7, 6.0 Hz, 2H), 7.44 (dd, J = 8.5, 2.6 Hz, 2H), and 4.31 (s, 4H). ^13^C-NMR (100 MHz, CDCl3): δ (ppm) 43.75 (C-24, C-25), 115.67 (C-11, C-13), 119.11 (C-3, C-14), 121.11 (C-9, C-18), 125.91 (C-1, C-8), 129.78 (C-10, C-16), 132.85 (C-21, C-23), 137.63 (C-9, C-19), 163.97 (C-4, C-15), and 182.60 (C-12). HRMS (ESI) *m*/*z*: 469.9391 [M+H]+, calcd. for C_19_H_13_Br_2_F_2_, NO 469.9391. ChemDraw-predicted melting point: 732.68.

##### Synthesis of 3,5-Bis(2-bromo-4-chlorobenzyl)-4-piperidone (2Br-4Cl, New Derivative)

There was a peak at 9.915 min under 324 nm. There was a light-yellow powder yield of 82.24%. ^1^H-NMR (400 MHz, DMSO): δ (ppm) 10.32 (s, 1H), 7.97 (d, J = 2.0 Hz, 1H), 7.86 (s, 2H), 7.64 (dd, J = 8.4, 2.1 Hz, 1H), 7.52 (d, J = 8.3 Hz, 1H), 4.31 (s, 1H), and 2.55–2.48 (m, 1H). ^13^C-NMR (100 MHz, CDCl3): δ (ppm) 48.53 (C-1, C-17), 130.57 (C-11, C-22), 133.40 (C-3, C-16), 135.10 (C-12, C-24), 137.06 (C-14, C-23), 137.74 (C-11, C-22), 140.41 (C-7, C-13), 142.29 (C-5, C-6), and 187.26 (C-9). HRMS (ESI) *m*/*z*: 501.8800 [M+H]+, calcd. for C_19_H_13_Br_2_Cl_2_, NO 501.8800. ChemDraw-predicted melting point: 791.34.

##### Synthesis of 3,5-Bis(2-bromo-5-chlorobenzyl)-4-piperidone (2Br-5Cl, New Derivative)

At 8.757 min under 303 nm, a strong absorption, the peak area accounted for more than 99% of the total peak area. There was a light-yellow powder yield of 79.29%. ^1^H-NMR (400 MHz, DMSO): δ (ppm) 10.28 (s, 1H), 7.86–7.83 (m, 2H), 7.81 (d, J = 8.5 Hz, 2H), 7.62 (d, J = 2.5 Hz, 2H), 7.51 (dd, J = 8.6, 2.6 Hz, 2H), and 4.35 (m, 4H). ^13^C-NMR (100 MHz, CDCl3): δ (ppm) 43.68 (C-13, C-14), 123.23 (C-18, C-19), 130.42 (C-1, C-7), 130.72 (C-6, C-10), 131.67 (C-8, C-9), 133.14 (C-2, C-11), 135.09 (C-3, C-16), 135.90 (C-12, C-22), 137.53 (C-15, C-21), and 182.50 (C-5). HRMS (ESI) *m*/*z*: 501.8803 [M+H]+, calcd. for C_19_H_13_Br_2_Cl_2_, NO 501.8803. ChemDraw-predicted melting point: 791.34.

##### Synthesis of 3,5-Bis(3,5-dibromo-benzyl)-4-piperidone (3,5-2Br, New Derivative)

Under 320 nm, there was a strong absorption at 10.559 min, and the peak area accounted for more than 95% of the total peak area. There was a light-yellow powder yield of 82.93%. ^1^H-NMR (400 MHz, DMSO): δ (PPM) 9.95 (s, 1H), 7.97 (s, 2H), 7.79 (s, 4H), 7.58 (s, 2H), and 4.47 (s, 4H). ^13^C-NMR (100 MHz, CDCl3): δ (ppm) 43.98 (C-12, C-15), 123.32 (C-5, C-7, C-11, C-23), 130.65 (C-8, C-25), 132.18 (C-4, C-6, C-21, C-24), 134.88 (C-20, C-22), 136.91 (C-13, C-16), 138.18 (C-18, C-19), and 182.53 (C-14). HRMS (ESI) *m*/*z*: 591.7761 [M+H]+, calcd. For C_19_H_13_Br_4_, NO 591.7761. ChemDraw-predicted melting point: 851.1.

##### Synthesis of 3,5-Bis(4-bromo-2-fluorobenzyl)-4-piperidinone (4Br-2F, New Derivative)

At 8.362 min under 330 nm, there was a strong absorption, and the peak area accounted for more than 95% of the total peak area. There was a light-yellow powder yield of 88.66%. ^1^H-NMR (400 MHz, DMSO): δ (PPM) 10.35 (s, 1H), 7.93 (s, 2H), 7.81–7.73 (dd, J = 8.4, 1.9 Hz, 2H), 7.60 (dd, J = 8.3, 1.8 Hz, 2H), 7.48 (t, J = 8.2 Hz, 2H), and 4.32 (s, 4H). ^13^C-NMR (100 MHz, CDCl3): δ (ppm) 44.11 (C-1, C-4), 120.09 (C-9, C-20), 121.48 (C-13, C-16), 124.60 (C-10, C-22), 128.60 (C-8, C-24), 131.07 (C-7, C-14), 132.76 (C-3, C-6), 159.21 (C-17, C-25), and 182.33 (C-5). HRMS (ESI) *m*/*z*: 469.9374 [M+H]+, calcd. For C_19_H_13_Br_2_F_2_, NO 469.9374. ChemDraw-predicted melting point: 732.68.

##### Synthesis of 3,5-Bis(5-bromo-2-chlorobenzyl)-4-piperidone (5Br-2Cl, New Derivative)

At 8.763 min under 303 nm, a strong absorption, the peak area accounted for more than 97% of the total peak area. There was a light-yellow powder yield of 76.89%. ^1^H-NMR (400 MHz, DMSO): δ (ppm) 9.10 (s, 1H), 7.80 (s, 2H), 7.74–7.66 (m, 4H), 7.63–7.56 (m, 2H), 4.24 (m, 4H). ^13^C-NMR (100 MHz, CDCl3): δ (ppm) 44.73 (C-2, C-17), 120.68 (C-12, C-14), 132.15 (C-9, C-19), 133.02 (C-1, C-15), 133.23 (C-11, C-16), 133.59 (C-18, C-20), 133.82 (C-4, C-7), 134.29 (C-5, C-13), 134.65 (C-6, C-8), 183.75 (C-10). HRMS (ESI) *m*/*z*: 501.8797 [M+H]+, calcd. for C_19_H_13_Br_2_Cl_2_, NO 501.8797. ChemDraw predicted melting point: 791.34.

### 3.2. Anti-Tumour Activity Study

#### 3.2.1. Experimental Materials and Instruments

Human liver cancer cell line (HepG2, ATCC^®^ HB-8065™), human cervical cancer cell line (HeLa, ATCC^®^ CCL-2™), human breast cancer cell line (MCF-7, ATCC^®^ HTB-22™), human non-small cell lung cancer cell line (A549, ATCC^®^ CCL-185), and human normal liver cell line (L02, ATCC^®^ CRL-1417) were all purchased from Peking Union Medical College cell Bank; fetal bovine serum (Batch number: 2022057), PBS buffer (Batch number: 2042168), DMEM High sugar culture medium (Batch number: 2042166), and DMEM Low sugar culture medium (Batch number: 2030101) were all purchased from Biological Industries Tel Aviv, Israel; the 0.25% trypsin EDTA (Batch number: CR2012104), Penicillomycin mixture (Batch number: HJ202305), Annexin V and PI Apoptosis Detection Kit (Batch number: HY0821, Manufacturer: Biorigin (Beijing, China) Inc.), RIPA lysate (Batch number: G2002), GAPDH (Batch Number: GB12002), Electrophoresis buffer (Batch number: G2018), and TBS buffer (Batch number: G0001-2L) were all purchased from Wuhan Servicebio Technology Co., Ltd Wuhan, China.; Dimethyl sulfoxide DMSO (Batch number: Z28N11Y132686, Manufacturer: Shanghai Yuanye Biology Shanghai, China); MTT (Batch number: 19110601), and Cell Counting Kit-8 (Batch Number: DCM7126) were all purchased from Beijing Lablead Trading Co., Ltd., Beijing, China.; Cell culture flask (25 cm^2^) and 6, 24, 96-well plate were all purchased from American Corning Incorporat., Corning, NY, USA.

Blood counting board was purchased from Beijing Aobox Biology Technology Co., Ltd., Beijing, China; carbon dioxide cell incubator and Sorall ST 8R High speed refrigerated centrifuge were all purchased from American Thermo Fisher; multifunctional microplate reader was purchased from Finnish Thermoelectric Multiskan GO; purification table was purchased from Shanghai Boxun Medical Biological Instrument Co., Ltd., Shanghai, China; JYC-200B air shaker was purchased from Shanghai Jinwen Instrument equipment Co., Ltd., Shanghai, China; FA1204B precision electronic balance was purchased from Shanghai Precision Instrument Co., Ltd., Shanghai, China; Zebrafish incubator was purchased from Changzhou Xinhua Electronics Co., Ltd., Changzhou, China; Jy92-11n Ultrasonic cell breaker was purchased from Ningbo Xinzhi Biology Ningbo, China; inverted microscope, fluorescence inverted microscope, and SMZ18 Stereo microscope were all purchased from Japan Nikon Corporation.

#### 3.2.2. Inhibition of Four Kinds of Tumour Cells

##### MTT Test [46]

The tumour cell suspension in logarithmic growth phase, containing about 5 × 10^3^ cells, was taken and seeded onto a 96-well plate and gently shaken to disperse the cells evenly at the bottom of the plate. The cells were incubated in a constant temperature (37 °C) incubator for 24 h. The old medium was discarded, and 100 μL of the prepared working solution of piperidinone monocarbonyl curcumin analogues at different concentrations was added, and the cells were incubated in a constant temperature incubator for 24 h. Blank medium wells, blank cell wells, and 0.9% DMSO (highest content) wells were set up, with 6 multi-well and 3 multi-plates for each concentration. The discarded old medium was absorbed, and 100 μL of 10% 5 mg/mL MTT working solution was added to each well (operated away from light) and placed in a cell incubator for further incubation for 4 h. The old medium was discarded, and 100 μL of DMSO was added to each well and shaken in an air shaker at 100 RPM for 10 min. The absorbance of the OD value was measured at 490 nm by a multifunctional microplate analyzer. According to Formula (1), the inhibition rates of different piperidones monocarbonyl curcumin analograms on 4 tumour cells were calculated, and IC_50_ was calculated by GraphPad Prism 8.0:

Inhibition rate (%) = (OD _blank cell group_ − OD _administration group_)/OD _blank cell group_ (1)

##### Growth Curve of A549 Cells Induced by Five Promising Compounds [47]

A549 cells in the logarithmic growth phase and in a good growth state were collected. After routine digestion and centrifugation, the cells were diluted with DMEM low-glucose complete medium, suspended in a single cell state, and inoculated in 96-well plates. Each well was inoculated with 100 μL cell suspension containing 5 × 10^3^ cells. The 96-well plate was shaken to disperse the cells evenly at the bottom of the cell orifice plate, and the cells were cultured in the cell incubator for 24 h. The old medium was discarded, complete medium containing different concentrations of liquid medicine was added, and the culture continued in the cell culture incubator. At 4, 6, 12, 24, 48, and 72 h, the old medium was discarded and 100 μL of medium containing 10% CCK-8 was added. The medium was placed in the cell incubator and continued to incubate for 2 h. The OD value was measured at 450 nm with an enzyme calibration and the inhibition rate of different compounds at different concentrations was calculated. GraphPad Prism 8.0 was used to plot the inhibition rate of cell growth at different concentrations of the five major compounds. The drug duration curves were plotted with time as the X-axis and inhibition rate as the Y-axis.

##### The Toxicity of Five Promising Compounds to Human Normal Hepatocytes (L02) [48]

The cells were passaged to the fourth generation of L02 cells, and the cell suspension containing about 5 × 10^3^ cells was added to each well of the 96-well plate and cultured in a cell culture incubator for 24 h. The old medium was discarded, and different concentrations of liquid medicine (6 porous and 3 plates for each concentration) were added and incubated for 24 h. The old medium was discarded, 10% of 5 mg/mL MTT working solution was added in the dark, and the cells were incubated in a cell incubator for 4 h. The old medium was discarded, 100 μL DMSO was added to each well, and 100 RPM was oscillated on an air shaker for 10 min. The OD value was measured at 490 nm with a multifunctional microplate reader. According to the OD value, the inhibition rate of different piperidone monocarbonyl curcumin analogues on L02 cells was calculated according to Formula (1), and IC_50_ was calculated by Graph Pad Prism 8.0.

##### Effects of Five Promising Compounds on A549 Cell Migration [49]

A549 cells in logarithmic phase were digested with 0.25% trypsin and blown to single cell suspension with a dropper. The cell density was adjusted to 5 × 10^5^ cells/mL. The cells were inoculated on 6-well plates, 2 mL per well, and cultured in a cell incubator at 37 °C, 5% CO_2_, and saturated humidity for 24 h until the gaps were basically fused. A 10 μL micro pipette tip was used to scratch vertically in a 6-well plate. After washing with PBS solution 3 times, the drug-containing medium was added, 2 mL per well, and three wells were set in each group. The culture plates were placed in a 5% CO_2_ incubator at 37 °C for 6 h and 24 h and observed and photographed with an inverted fluorescence microscope. The images were obtained by NIS Elements Viewer 4.2.0, and the scratch area and width were calculated. The cell migration rate was calculated by Formula (2):

[Cell migration rate (%) = (0 h scratch area − scratch area at different times)/0 h scratch area × 100%](2)

##### A549 Cell Death Induced by Five Promising Compounds [50]

The cells were inoculated in a 6-well plate, and about 1 × 10^6^ A549 cells in the logarithmic growth phase were inoculated in each well. After the cells were cultured in a constant temperature incubator for 24 h, the old medium was discarded, and five kinds of piperidone monocarbonyl curcumin analogue solution (5, 10, and 20 μmol/L), curcumin (10, 20, and 40 μmol/L), and 0.9% DMSO were added as the blank control group for 24 h, and then the cell morphology was observed by inverted microscope (200×).

##### Effects of Five Promising Compounds on Apoptosis of A549 Cells [51]

The A549 cells in logarithmic growth phase were inoculated in 6-well plates at 1 × 10^6^ cells. After resting in the incubator for 24 h, the cells were treated with different concentrations of drugs. The blank control group was added with the medium with the highest concentration of DMSO. After 24 h of culture, cells were collected, washed with PBS, transferred to a flow tube, and resuspended with 100 μL of 1 × Annexin V conjugate. Add 4 μL YF4/88-Annexin N and 5 μL PI working solution. The cells were incubated in the dark at room temperature for 10 min, transferred to a flow cytometer tube, added with 200 μL of 1 × Annexin V conjugate, and immediately detected by flow cytometry.

##### Western Blot Was Used to Detect the Effects of Five Promising Compounds on the Expression Levels of Apoptosis-Related Proteins in A549 Cells [52,53]

A549 cells in logarithmic growth phase were seeded in 6-well plates at 1 × 10^6^ cells/mL per well. The cells were cultured overnight in an incubator. Different concentrations of drug-containing medium of 2,5-2Cl, 2Br-5Cl, 2-Cl, 2-F, benzaldehyde (1.25, 2.5, 5 μmol/L), curcumin (20 μmol/L), and cisplatin (30 μmol/L) were added, and the blank control group with the highest concentration of DMSO was set up and the culture was continued for 48 h. The RIPA cell lysis buffer containing protease inhibitors was added to the Petri dish to collect the proteins of each experimental group, and the supernatant was collected as a protein sample after ultrasonic centrifugation. Protein quantification was performed by the BCA method. A total of 20 μg protein was separated by 10% SDS-PAGE, and then the protein was transferred to a PVDF membrane. The membrane was blocked with 5% skim milk for 1 h, and the corresponding primary antibody was incubated at 4 °C overnight. After washing the membrane three times with TBST, the bands were incubated with the corresponding secondary antibodies at room temperature for 1 h. After cleaning the membrane with TBST 3 times, the darkroom was developed with ECL luminescence. Using GAPDH as an internal reference, the protein bands were analyzed by ImageJ 1.8.0.

### 3.3. Study on Antibacterial Effect

#### 3.3.1. Experimental Materials and Instruments

Nutritional Broth and Nutritional AGAR (Beijing Aoboxing Biotechnology Co., Ltd., Beijing, China); Dimethyl sulfoxide DMSO (Batch Number: Z28N11Y132686, Manufacturer: Shanghai Yuanye Biology Shanghai, China); penicillin (Batch Number: A8180,) and tobramycin (Batch Number: T8810) were all purchased from Beijing Solaibao Biotechnology Co., Ltd.Beijing, China; multidrug-resistant *Staphylococcus aureus* (MRSA. Batch Number: 19XTH0119, Manufacturer: Dongzhimen Hospital, Beijing University of Chinese Medicine).

High-pressure steam sterilization pot (Shanghai Boxun Industrial Co., Ltd., Shanghai, China); multifunctional microplate reader (Finnish hot spot Multiskan GO); HFease 1800 Biosafety cabinet (Shanghai Likang Precision Technology Co., Ltd., Shanghai, China); constant temperature incubator (Chongqing Ruili Electronic Instrument Equipment Co., Ltd., Chongqing, China); table low-speed centrifuge (Jingli BRAND LD5-2B); Ultra-low temperature refrigerator (American Thermo Scientific, Waltham, MA, USA); FA1204B Electronic analytical balance (Shanghai Precision Instrument Co., Ltd., Shanghai, China); BT125D electronic analytical balance (Beijing Sartorius Instrument System Co., Ltd., Beijing, China).

#### 3.3.2. Determination of Minimum Inhibitory Concentration (MIC) [54,55]

Firstly, the inhibition rates of all compounds against MRSA and *S. ureus* at the maximum concentration of 160 μmol/L were determined by the microbroth dilution method, and the results are shown in Formula (3). Then, the MIC values of 100% compounds at the maximum inhibitory concentration were obtained by the broth microdilution method. The specific experiments are as follows: First, in the 96-well plate, add 100 μL of compound working solution from column 2 to column 8, with concentrations decreasing from high to low, repeating each concentration 6 times. Add 100 μL of NB nutrient broth containing 1% DMSO to column 9, and 100 μL of blank NB nutrient broth to columns 10 and 11. Then, add 10 μL of bacterial suspension to each well from columns 2 to 10. Place the 96-well plate in a 37 °C incubator for 16 h at 100 rpm and measure the absorbance at 600 nm by microplate reader. Columns 2 to 9 represent the drug treatment group, column 10 is the negative control group, and column 11 is the blank control group. The minimum concentration that inhibits bacterial growth is the MIC value of the compound:

Bacterial inhibition rate (%) = (OD_ administration _ − OD _control_)/(OD_control_ − OD _blank_) (3)

#### 3.3.3. The Inhibition Curve of Promising Compounds Against MRSA

Based on the results of previous experiments, the promising compounds were selected. According to the administration method under ‘2.3.2’, the absorbance values of the promising compounds at 600 nm at different concentrations of bacteria at 4, 14, 18, and 24 h were measured by a microplate reader, and the inhibitory effect of the compounds on MRSA at different time points was calculated. The time inhibition curve of the compound against MRSA was made with the abscissa as the time and the ordinate as the bacterial inhibition rate.

#### 3.3.4. Determination of Inhibition Zone of Promising Compounds [56]

Based on the results of previous experiments, the promising compounds were selected, and the size of the inhibition zone at 160 μmol/L was determined. The specific operation was as follows: The nutrient agar medium, after high temperature sterilization, was poured into about 20 mL per 9 cm plate in an ultra-clean bench and naturally cooled and solidified. Each plate was evenly coated with 100 μL MRSA bacterial suspension. The filter paper of 6 mm diameter soaked in the drug solution was dried by flame evaporation, and four tablets were placed in parallel into each plate, cultured in a constant temperature incubator at 37 °C for 16 h, and the size of the inhibition zone was measured.

### 3.4. Molecular Docking [57]

The 2D structure of the ligand was drawn by ChemDraw Professional 21.0, and the 3D structure was drawn by Chem3D 21.0. The MM2 module minimizes energy and saves the file. Then, we searched and downloaded the 3D structures of AKT2 [58,59] (PDB ID: 3D0E, 2.00 Å), ERK1 [60] (PDB ID: 4QTB, 1.40 Å), and PBP2a [42] (PDB ID: 1MWT, 2.45 Å) from the PDB (https://www.rcsb.org accessed on 17 November 2024) database. The receptors and ligands were routinely handled by PyMOL 2.4.0 and AutoDockTools 1.5.7 software, and the molecular docking of the treated receptors and ligands was carried out by AutoDock Vina. Finally, the docking file results of the target protein and ligand with the smallest binding energy were visualized in PyMOL.

### 3.5. ADMET Property Prediction [61]

ADMET refers to drug absorption (A), distribution (D), metabolism (M), excretion (E), and toxicity (T). The 2D compound structure was converted into SMILES format by ChemDraw Professional 21.0 and uploaded to SwissADME (https://www.swissadme.ch accessed on 17 November 2024), pkCSM (https://biosig.lab.uq.edu.au/pkcsm/prediction accessed on 17 November 2024), and admetSAR-2.0 (http://lmmd.ecust.edu.cn/admetsar2 accessed on 17 November 2024), three online web servers, to predict ADMET parameters and evaluate the pharmacokinetic characteristics and drug-like properties of the compounds.

Lipinski, Bioavailability Score, P-gp substrate, cytochrome P-450 1A2 (CYP1A2) inhibitor, CYP2C19 inhibitor, CYP2C9 inhibitor, CYP2D6 inhibitor, and CYP3A4 inhibitor were predicted by SwissADME. LogP, Total Clearance, Renal OCT2 substrate, AMES toxicity, hepatotoxicity, and Rat Oral Acute Toxicity (LD50) were predicted by pkCSM. Plasma protein binding and Nephrotoxicity were predicted by admetSAR-2.0.

### 3.6. Statistical Analysis [62]

The data were processed by statistical analysis software SAS 8.0 and GraphPad Prism 8.0, and the results were expressed as mean ± standard deviation. The data between the groups were compared by single-factor and two-factor analysis of variance. *p* < 0.05 was considered statistically significant, and *p* < 0.01 was considered extremely statistically significant.

## 4. Conclusions

In summary, 14 monocarbonyl curcumin piperidone compounds were synthesized by Claisen–Schmidt reaction in this study. The related physical and chemical properties of the analogues were characterized by proton nuclear magnetic resonance (^1^H-NMR) and carbon nuclear magnetic resonance (^13^C-NMR). The effects of analogues on the proliferation of A549 cells and the safety of normal human hepatocytes (L02) were detected by the MTT method and CCK-8 method. The effect of analogues on the migration of A549 cells was evaluated by the scratch wound healing test. The effect of the analogues on the apoptosis of A549 cells was evaluated by an inverted microscope and flow cytometry, and the effect of the analogues on the protein expression level in A549 cells was evaluated by Western blotting. Finally, the antibacterial activity of the analogues against MRSA was investigated by the broth microdilution method and filter paper method. In terms of network pharmacology, molecular docking was used to explore the interaction between analogues and AKT2, ERK1, and PBP2a action sites, and SwissADME, pkCSM, and admetSAR network servers were used to predict the pharmacokinetic characteristics and drug-like properties of analogues.

All the synthesized analogues have different degrees of anti-tumour and antibacterial dual activity. Among them, the newly synthesized analogues 2,5-2Cl and 2Br-5Cl had the strongest inhibitory effect on the proliferation and migration of A549 cells. 2,5-2Cl tended to regulate the down-regulation of AKT protein expression, and 2Br-5Cl tended to inhibit the expression of ERK protein and induce apoptosis. The analogues 2-Cl and 2-F are prominent because of their significant anti-MRSA effects, and their ability to inhibit AKT and ERK pathways in A549 cells is strong. In general, the lead compounds in the curcumin analogues synthesized in this study may be developed as potential anti-tumour and antibacterial dual therapeutic agents.

## Figures and Tables

**Table 1:** IC_50_ of fourteen piperidone monocarbonyl curcumin analogues on four types of tumour cell lines (μmol/L, $\bar{x}\pm s$, *n* = 3).

Compound	HepG2	A549	Hela	MCF-7
Cisplatin solution	27.73 ± 3.26	41.21 ± 3.49	59.44 ± 1.70	55.24 ± 4.09
Curcumin	17.63 ± 3.43	33.51 ± 3.83	29.17 ± 2.57	39.22 ± 2.81
Benzaldehyde	10.34 ± 1.88	4.66 ± 2.39	12.36 ± 2.91	5.88 ± 1.44
2-F (EF24)	6.15 ± 3.88	3.99 ± 1.19	21.95 ± 2.98	13.18 ± 1.26
2-Cl	8.53 ± 2.61	4.81 ± 1.69	6.61 ± 1.93	5.70 ± 1.21
2-Br	7.03 ± 3.24	16.58 ± 1.84	12.27 ± 2.67	-
3-Br	18.29 ± 3.44	-	-	22.51 ± 3.55
4-Br	7.15 ± 4.62	15.54 ± 2.35	-	19.65 ± 3.68
2,5-2Cl	9.66 ± 1.09	2.87 ± 2.09	6.62 ± 2.10	4.72 ± 1.48
3,4-2Cl	-	11.87 ± 4.64	-	-
2Br-4F	18.27 ± 4.38	21.82 ± 3.68	-	-
2Br-4Cl	27.29 ± 2.64	18.22 ± 3.15	23.42 ± 3.34	-
2Br-5Cl	8.42 ± 1.99	4.75 ± 1.51	6.24 ± 2.46	6.36 ± 1.28
3,5-2Br	-	-	12.85 ± 3.18	-
4Br-2F	16.39 ± 1.97	20.22 ± 4.15	18.65 ± 3.66	28.46 ± 2.91
5Br-2Cl	29.31 ± 2.94	14.23 ± 3.85	-	-

***Note:*** “-” represents IC_50_ > 40 μmol/L.

**Table 2:** IC_50_ values for five promising monocarbonyl curcumin analogues on human normal liver cells (μmol/L $\bar{x}\pm s$, *n* = 3).

Compound	IC_50_ (μmol/L)
Cisplatin solution	48.36 ± 3.83
Curcumin	30.40 ± 1.04
Benzaldehyde	52.36 ± 1.89
2-F (EF24)	62.03 ± 3.54
2-Cl	70.48 ± 1.85
2,5-2Cl	48.59 ± 3.59
2Br-5Cl	58.25 ± 2.79

**Table 3:** The diameter of inhibition zone of promising analogues against MRSA ($\bar{x}\pm s$, *n* = 3).

Drug	Diameter of Bacteriostatic Zone (mm)
DMSO	6.00 ± 0.00
Tobramycin	10.67 ± 0.58 **
Penicillin	8.67 ± 0.58 **
2-F	8.33 ± 0.47 **
2-Cl	14.67 ± 0.58 **

***Note*:** Compared with blank group (DMSO), ** represents *p* < 0.01.

## Data Availability

All our data is provided in the article.

## Supplementary Materials

The following supporting information can be downloaded at https://www.mdpi.com/article/10.3390/ijms262412179/s1.
