# Supplementary material for: Synthesis, Anti-Tumour, and Antibacterial Activities of Monocarbonyl Curcumin Analogues of Piperidones
Source: Int J Mol Sci. 2025 Dec 18;26(24):12179. doi: 10.3390/ijms262412179 (PMC12734371; doi:10.3390/ijms262412179)
Supplement: Supplementary file 1 [file ijms-26-12179-s001.zip › ijms-3450742-supplementary.pdf]

Supplementary Materials

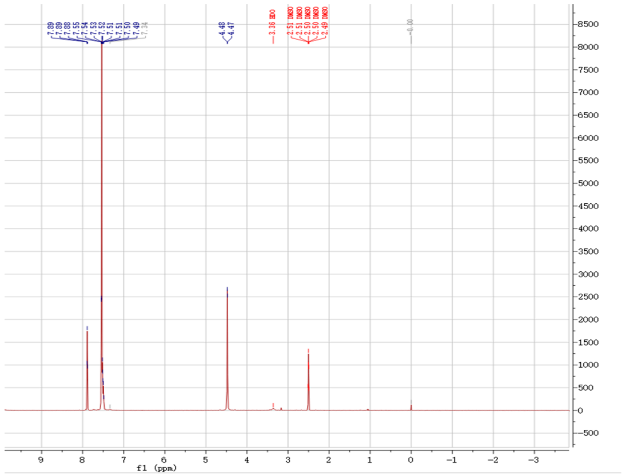

Figure S1 ^{1}H-NMR (400 MHz, DMSO-d6) spectrum of 3, 5-dibenzylidene-4-piperidone (Benzaldehyde)

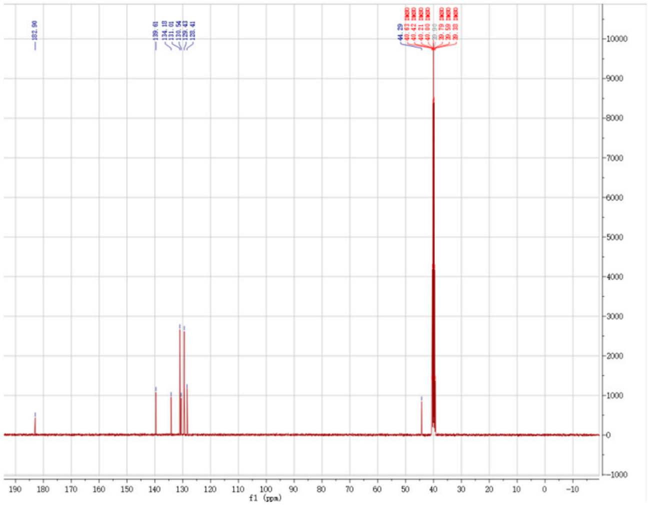

Figure S2 ^{13}C-NMR (100 MHz, $CDCl_3$) spectrum of 3, 5-dibenzylidene-4-piperidone (Benzaldehyde)

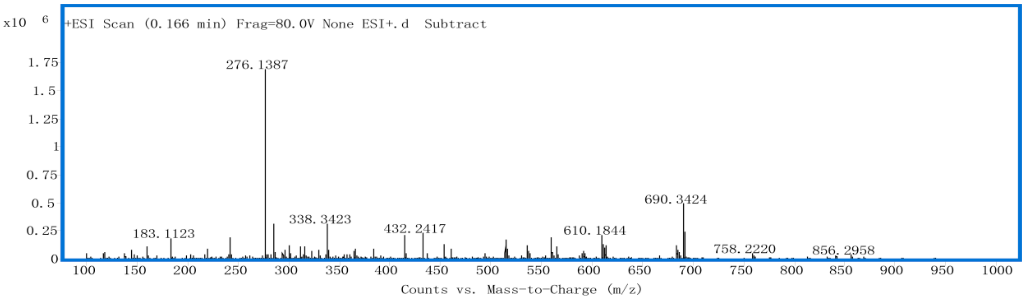

Figure S3 HR-MS (ESI) spectrum of 3, 5-dibenzylidene-4-piperidone (Benzaldehyde)

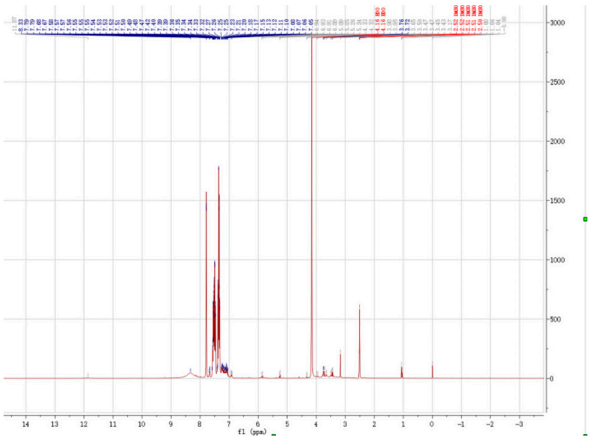

Figure S4 ^{1}H-NMR (400 MHz, DMSO-d6) spectrum of 3,5-bis(2-fluorobenzyl)-4-piperidinone (2-F)

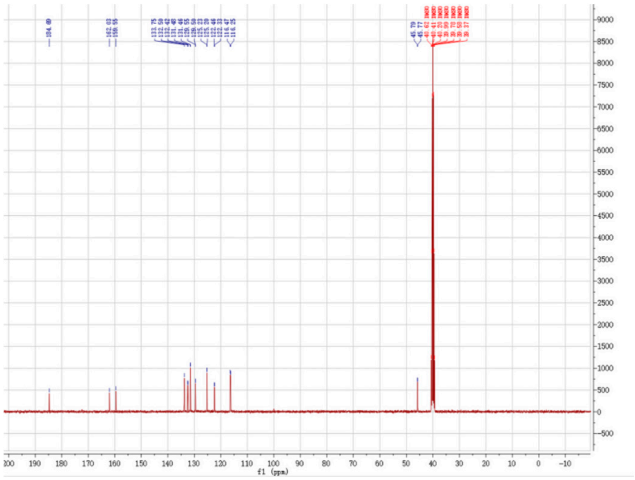

Figure S5 ^{13}C-NMR (100 MHz, $CDCl_3$) spectrum of 3,5-bis(2-fluorobenzyl)-4-piperidinone (2-F)

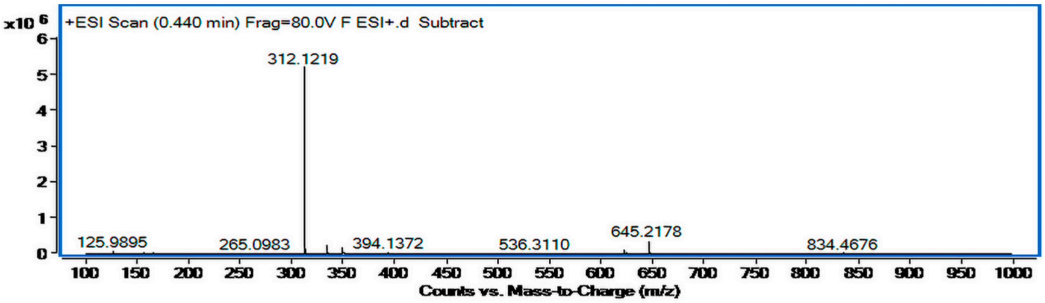

Figure S6 HR-MS (ESI) spectrum of 3,5-bis(2-fluorobenzyl)-4-piperidinone (2-F)

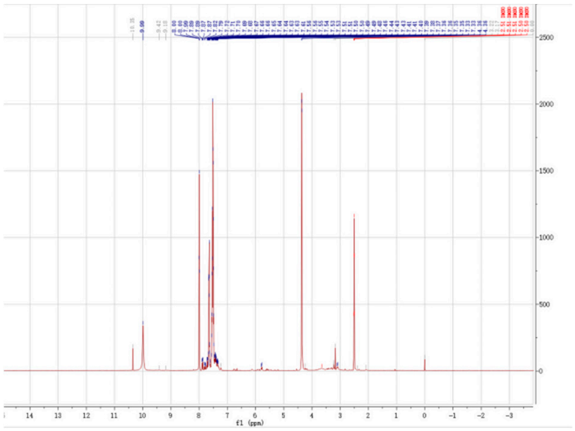

Figure S7 ^{1}H-NMR (400 MHz, DMSO-d6) spectrum of 3, 5-bis(2-chloro-benzyl)-4-piperidinone (2-Cl)

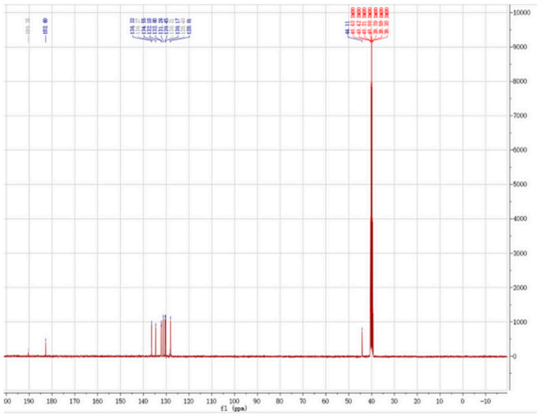

Figure S8 ^{13}C-NMR (100 MHz, $CDCl_3$) spectrum of 3, 5-bis(2-chloro-benzyl)-4-piperidinone (2-Cl)

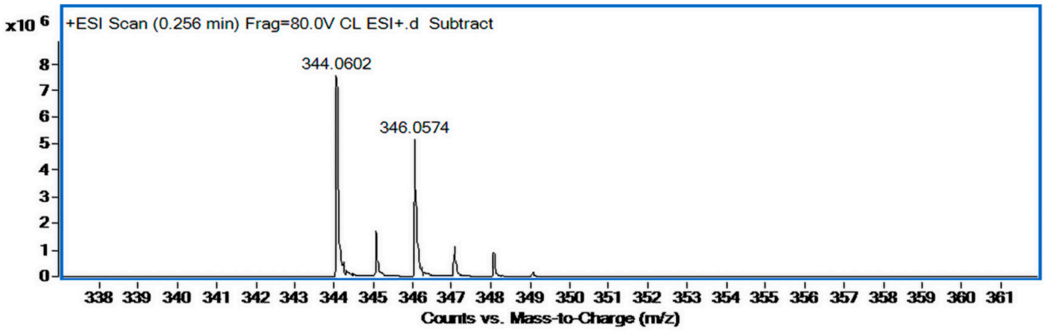

Figure S9 HR-MS (ESI) spectrum of 3, 5-bis(2-chloro-benzyl)-4-piperidinone (2-Cl)

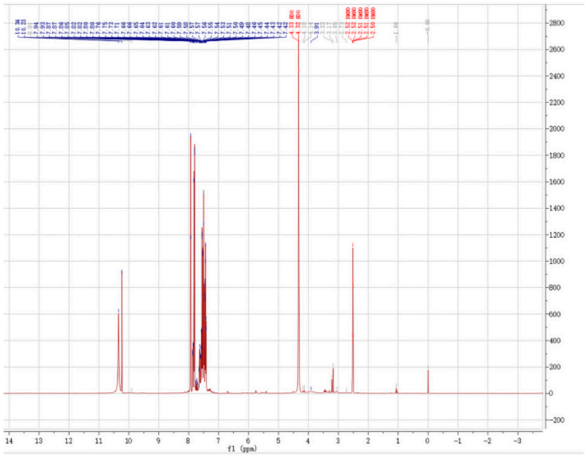

Figure S10 ^{1}H-NMR (400 MHz, DMSO-d6) spectrum of 3, 5-bis(2-bromo-benzyl)-4-piperidone (2-Br)

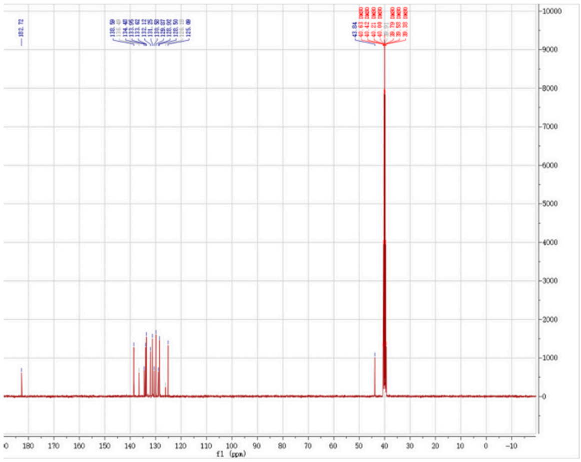

Figure S11 ^{13}C-NMR (100 MHz, $CDCl_3$) spectrum of 3, 5-bis(2-bromo-benzyl)-4-piperidone (2-Br)

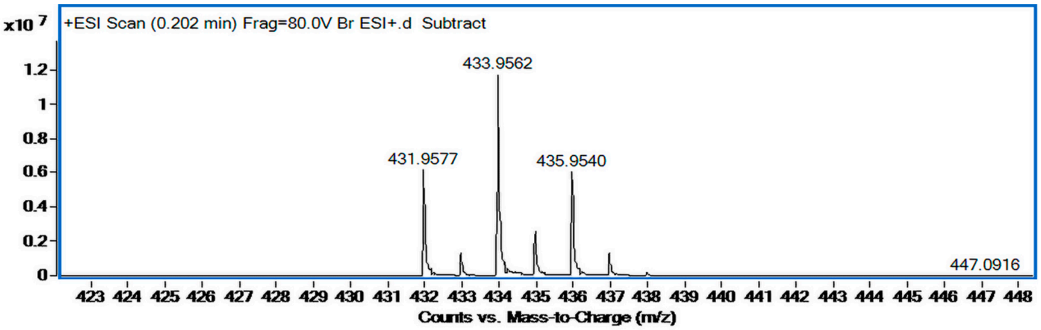

Figure S12 HR-MS (ESI) spectrum of 3, 5-bis(2-bromo-benzyl)-4-piperidone (2-Br)

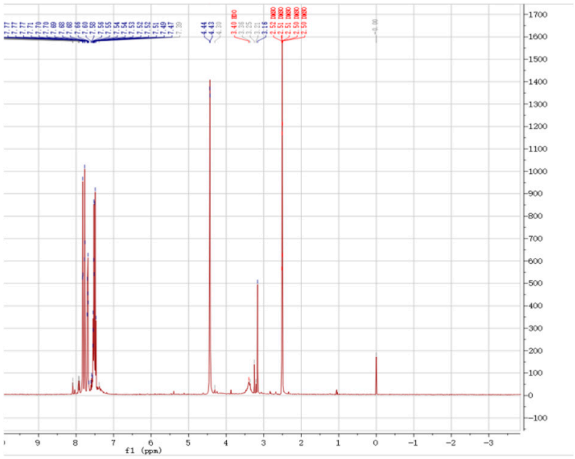

Figure S13 ^{1}H-NMR (400 MHz, DMSO-d6) spectrum of 3, 5-bis(3-bromo-benzyl)-4-piperidone (3-Br)

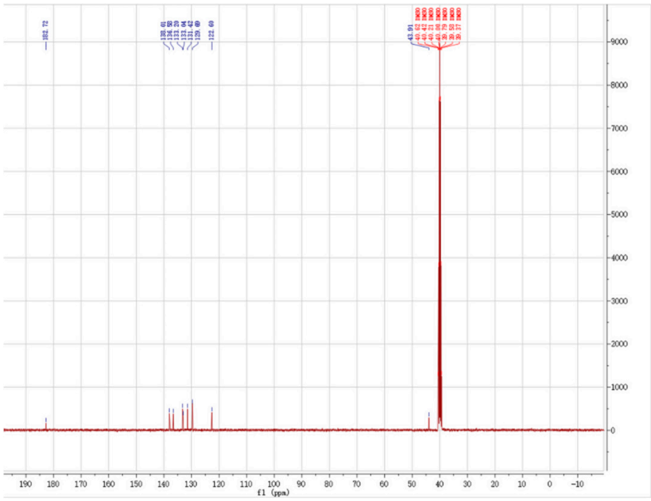

Figure S14 ^{13}C-NMR (100 MHz, $CDCl_3$) spectrum of 3, 5-bis(3-bromo-benzyl)-4-piperidone (3-Br)

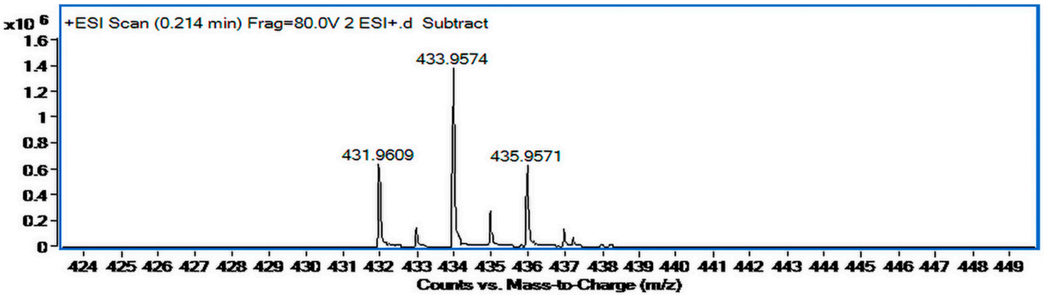

Figure S15 HR-MS (ESI) spectrum of 3, 5-bis(3-bromo-benzyl)-4-piperidone (3-Br)

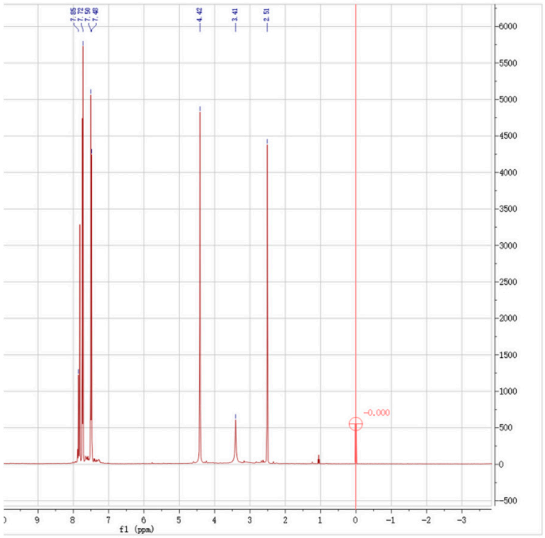

Figure S16 ^{1}H-NMR (400 MHz, DMSO-d6) spectrum of 3, 5-bis(4-bromo-benzyl)-4-piperidinone (4-Br)

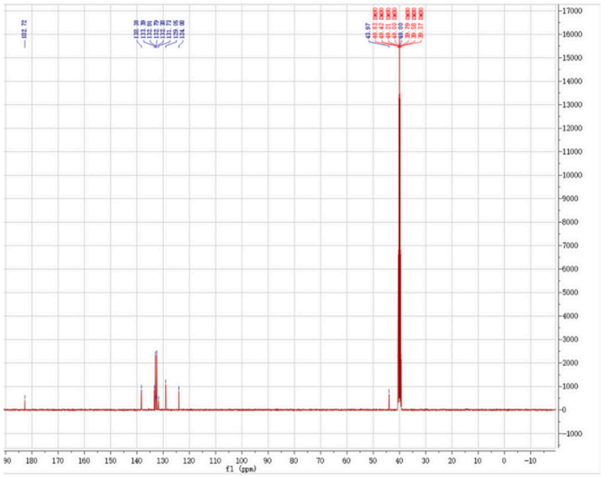

Figure S17 ^{13}C-NMR (100 MHz, $CDCl_3$) spectrum of 3, 5-bis(4-bromo-benzyl)-4-piperidinone (4-Br)

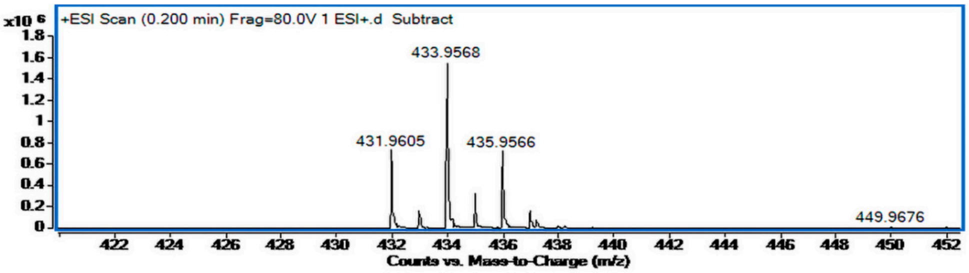

Figure S18 HR-MS (ESI) spectrum of 3, 5-bis(4-bromo-benzyl)-4-piperidinone (4-Br)

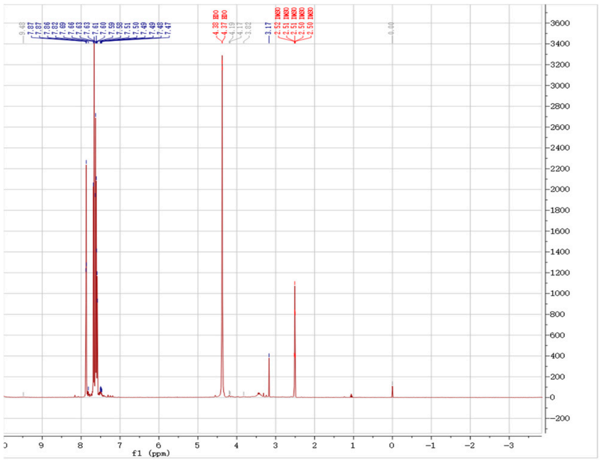

Figure S19 ^{1}H-NMR (400 MHz, DMSO-d6) spectrum of 3, 5-bis(2, 5-dichlorobenzyl)-4-piperidone (2,5-2Cl)

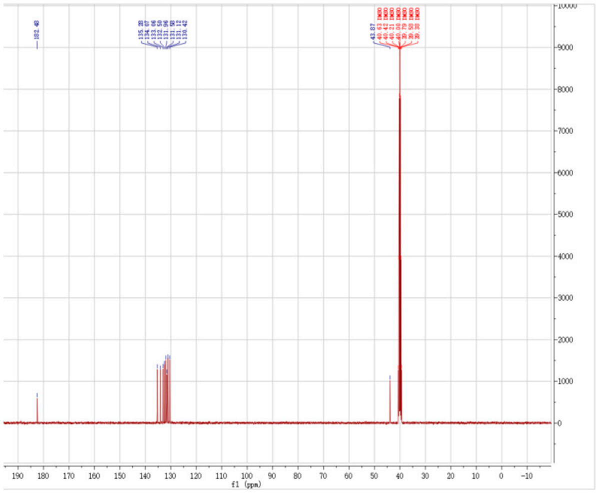

Figure S20 ^{13}C-NMR (100 MHz, $CDCl_3$) spectrum of 3, 5-bis(2, 5-dichlorobenzyl)-4-piperidone (2,5-2Cl)

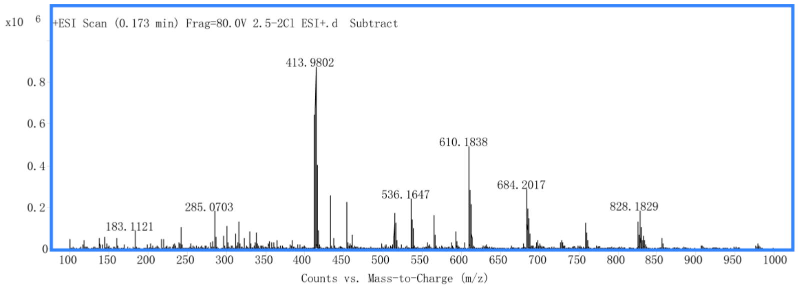

Figure S21 HR-MS (ESI) spectrum of 3, 5-bis(2, 5-dichlorobenzyl)-4-piperidone (2,5-2Cl)

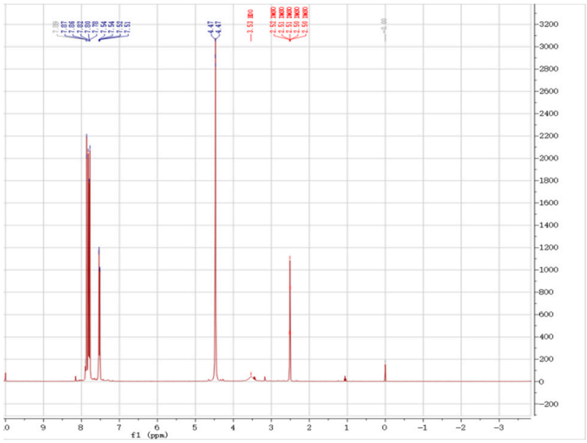

Figure S22 ^{1}H-NMR (400 MHz, DMSO-d6) spectrum of 3, 5-bis(3, 4-dichlorobenzylidene)-4-piperidone (3,4-2Cl)

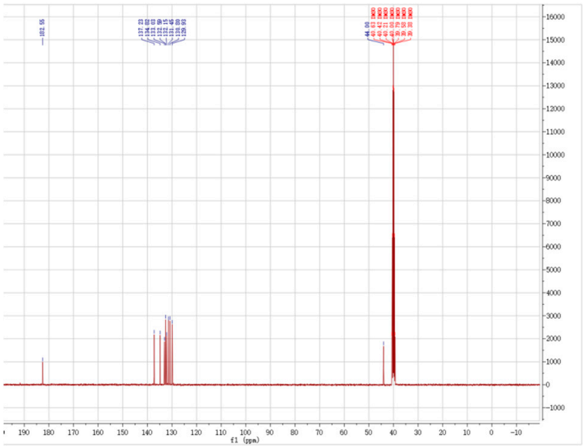

Figure S23 ^{13}C-NMR (100 MHz, $CDCl_3$) spectrum of 3, 5-bis(3, 4-dichlorobenzylidene)-4-piperidone (3,4-2Cl)

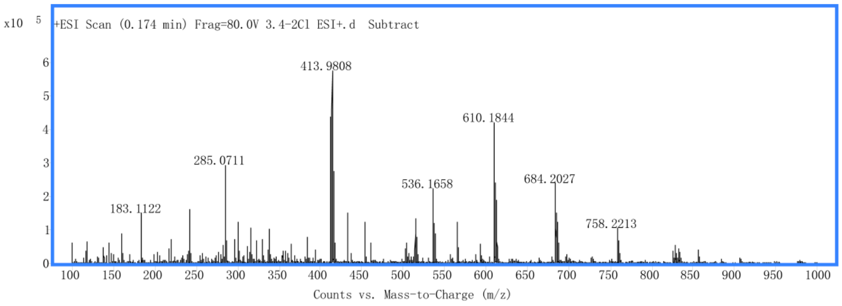

Figure S24 HR-MS (ESI) spectrum of 3, 5-bis(3, 4-dichlorobenzylidene)-4-piperidone (3,4-2Cl)

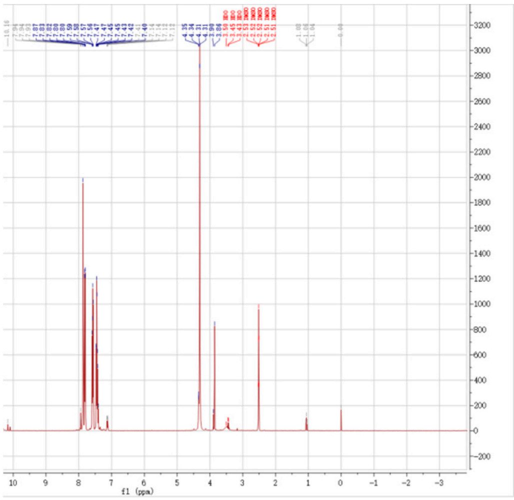

Figure S25 ^{1}H-NMR (400 MHz, DMSO-d6) spectrum of 3, 5-bis(2-bromo-4-fluorobenzyl)-4-piperidone (2Br-4F)

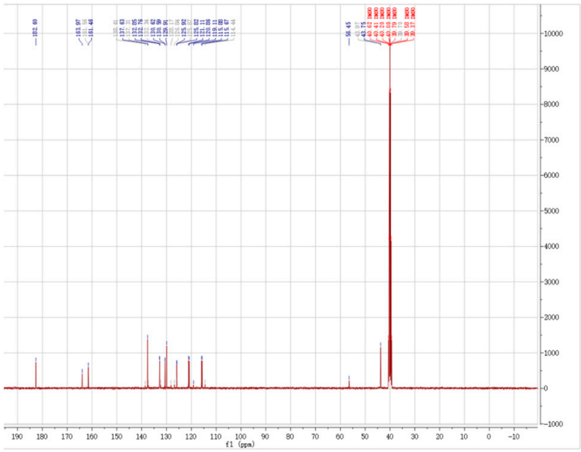

Figure S26 ^{13}C-NMR (100 MHz, $CDCl_3$) spectrum of 3, 5-bis(2-bromo-4-fluorobenzyl)-4-piperidone (2Br-4F)

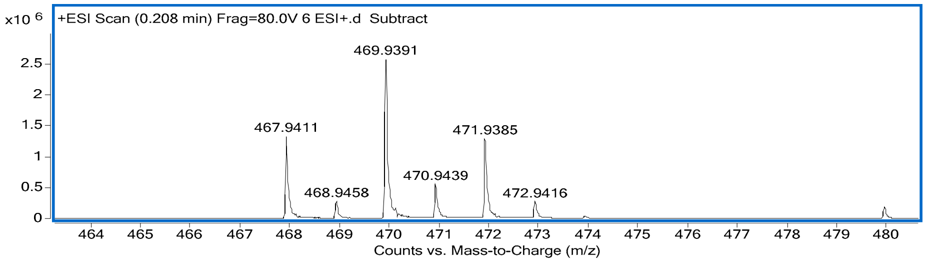

Figure S27 HR-MS (ESI) spectrum of 3, 5-bis(2-bromo-4-fluorobenzyl)-4-piperidone (2Br-4F)

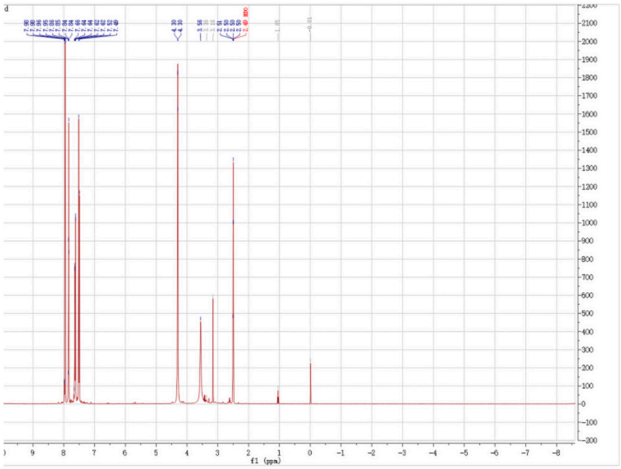

Figure S28 ^{1}H-NMR (400 MHz, DMSO-d6) spectrum of 3, 5-bis(2-bromo-4-chlorobenzyl)-4-piperidone (2Br-4Cl)

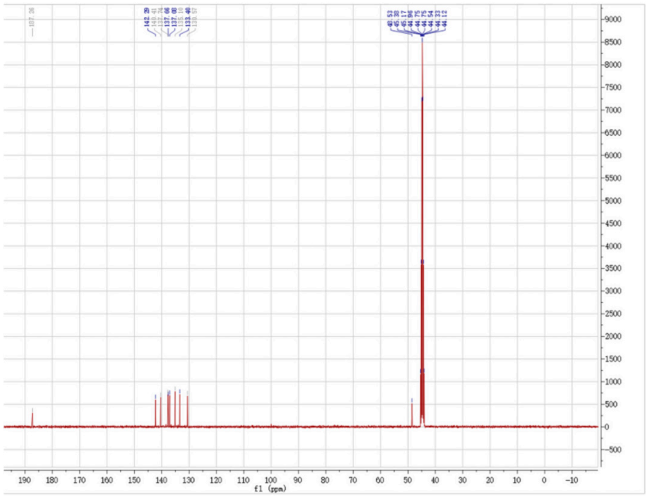

Figure S29 ^{13}C-NMR (100 MHz, $CDCl_3$) spectrum of 3, 5-bis(2-bromo-4-chlorobenzyl)-4-piperidone (2Br-4Cl)

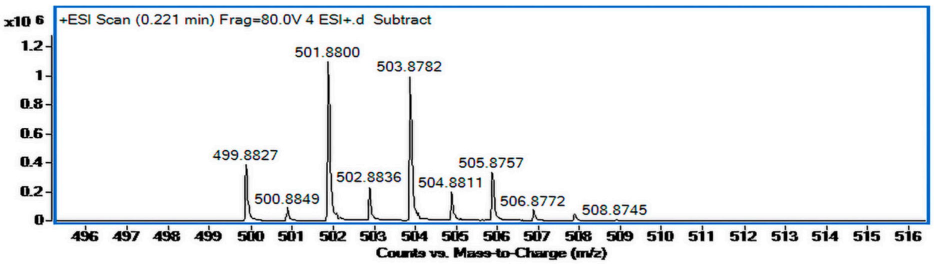

Figure S30 HR-MS (ESI) spectrum of 3, 5-bis(2-bromo-4-chlorobenzyl)-4-piperidone

(2Br-4Cl)

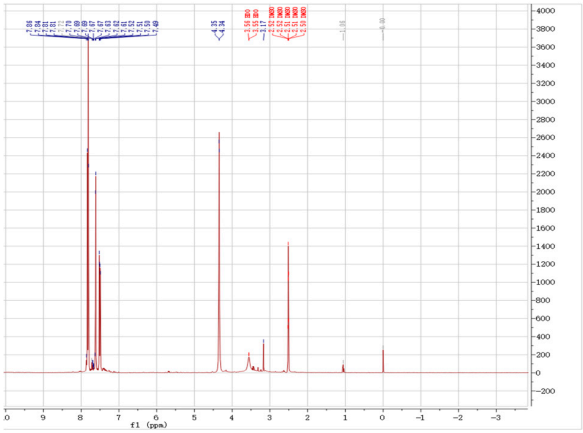

Figure S31 ^{1}H-NMR (400 MHz, DMSO-d6) spectrum of 3, 5-bis(2-bromo-5-chlorobenzyl)-4-piperidone (2Br-5Cl)

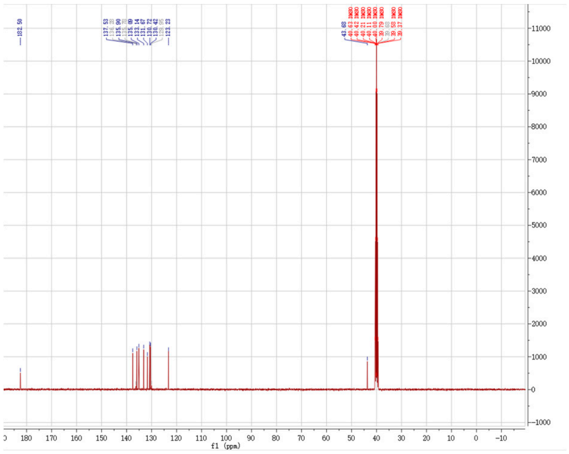

Figure S32 ^{13}C-NMR (100 MHz, $CDCl_3$) spectrum of 3, 5-bis(2-bromo-5-chlorobenzyl)-4-piperidone (2Br-5Cl)

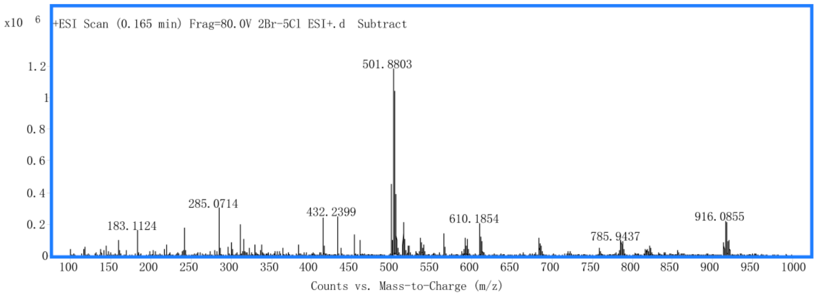

Figure S33 2Br-5Cl HR-MS (ESI) spectrum of 3, 5-bis(2-bromo-5-chlorobenzyl)-4-piperidone

(2Br-5Cl)

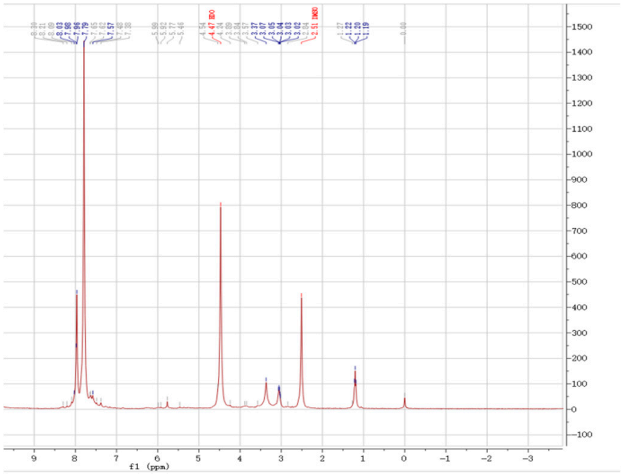

Figure S34 ^{1}H-NMR (400 MHz, DMSO-d6) spectrum of 3, 5-bis(3, 5-dibromo-benzyl)-4-piperidone (3,5-2Br)

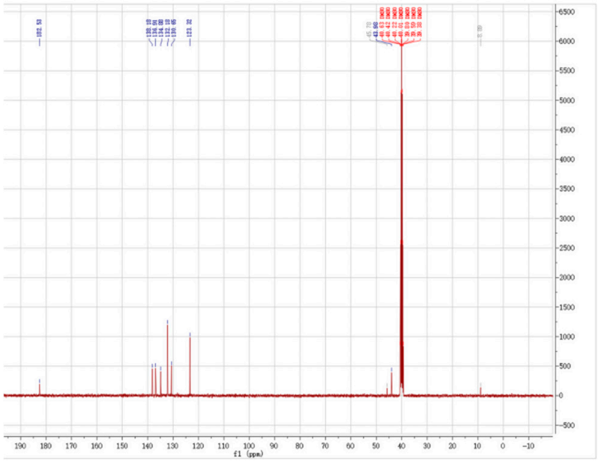

Figure S35 ^{13}C-NMR (100 MHz, $CDCl_3$) spectrum of 3, 5-bis(3, 5-dibromo-benzyl)-4-piperidone (3,5-2Br)

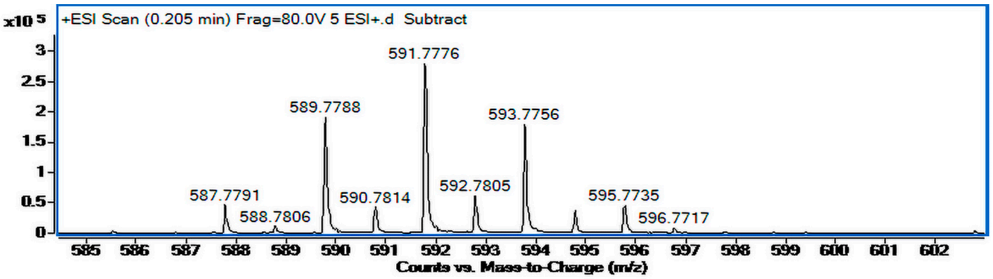

Figure S36 HR-MS (ESI) spectrum of 3, 5-bis(3, 5-dibromo-benzyl)-4-piperidone (3,5-2Br)

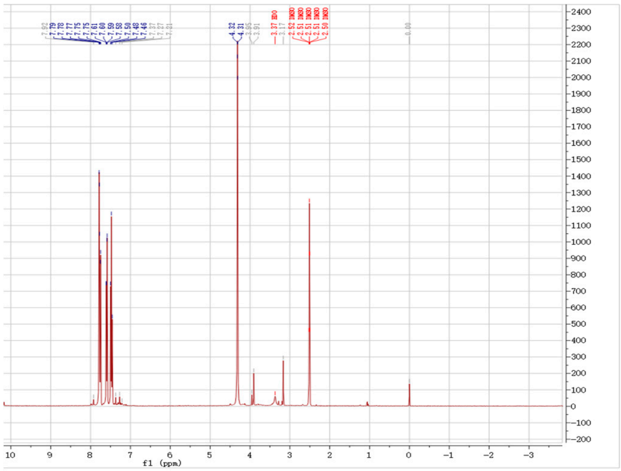

Figure S37 ^{1}H-NMR (400 MHz, DMSO-d6) spectrum of 3, 5-bis(4-bromo-2-fluorobenzyl)-4-piperidinone (4Br-2F)

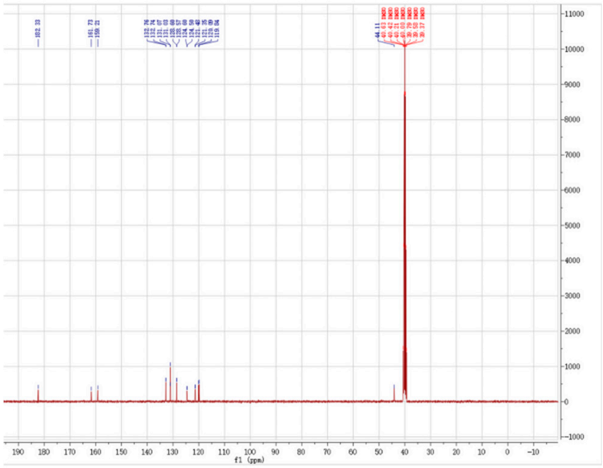

Figure S38 ^{13}C-NMR (100 MHz, $CDCl_3$) spectrum of 3, 5-bis(4-bromo-2-fluorobenzyl)-4-piperidinone (4Br-2F)

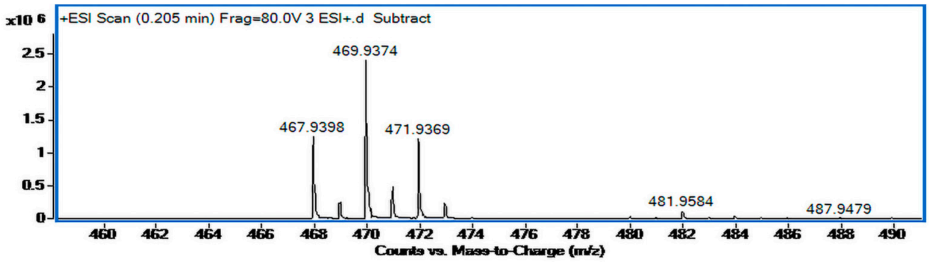

Figure S39 HR-MS (ESI) spectrum of 3, 5-bis(4-bromo-2-fluorobenzyl)-4-piperidinone

(4Br-2F)

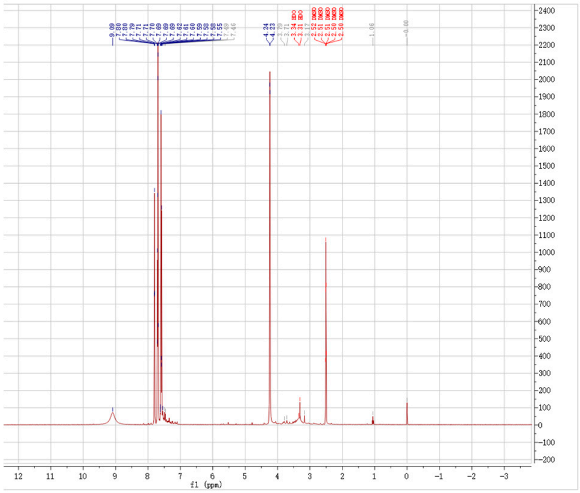

Figure S40 ^{1}H-NMR (400 MHz, DMSO-d6) spectrum of 3,5-bis(5-bromo-2-chlorobenzyl)-4-piperidone (5Br-2Cl)

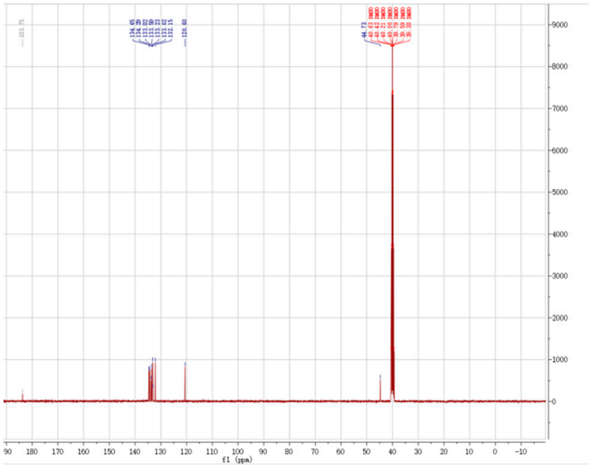

Figure S41 ^{13}C-NMR (100 MHz, $CDCl_3$) spectrum of 3,5-bis(5-bromo-2-chlorobenzyl)-4-piperidone (5Br-2Cl)

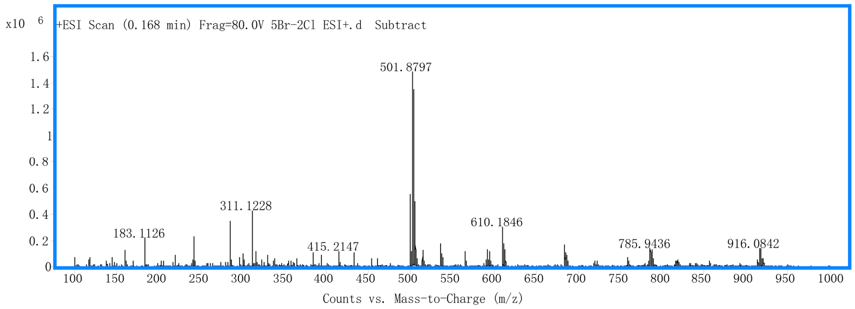

Figure S42 HR-MS (ESI) spectrum of 3, 5-bis(5-bromo-2-chlorobenzyl)-4-piperidone (5Br-2Cl)
